# MYCN expression induces replication stress and sensitivity to PARP inhibition in neuroblastoma

**DOI:** 10.18632/oncotarget.27329

**Published:** 2020-06-09

**Authors:** David King, Xiao Dun Li, Gilberto S. Almeida, Colin Kwok, Polly Gravells, Daniel Harrison, Saoirse Burke, Albert Hallsworth, Yann Jamin, Sally George, Simon P. Robinson, Christopher J. Lord, Evon Poon, Daniel Yeomanson, Louis Chesler, Helen E. Bryant

**Affiliations:** ^1^ Academic Unit of Molecular Oncology, Sheffield Institute for Nucleic Acids (SInFoNiA), Department of Oncology and Metabolism, University of Sheffield, Sheffield, UK; ^2^ Divisions of Clinical Studies and Cancer Therapeutics, The Institute of Cancer Research, Sutton, UK; ^3^ Divisions of Radiotherapy & Imaging, The Institute of Cancer Research, Sutton, UK; ^4^ The Children and Young People’s Unit, The Royal Marsden NHS Trust, Sutton, UK; ^5^ CRUK Gene Function Laboratory and Breast Cancer Now Research Centre, The Institute of Cancer Research, London, UK; ^6^ Sheffield Children’s Hospital, Western Bank, Sheffield, UK; ^#^ Present address: Medical Research Council Cancer Unit, Hutchison/Medical Research Council Research Centre, University of Cambridge, Cambridge, UK

**Keywords:** PARP inhibitor, neuroblastoma, MYCN, replication stress, replication fork stalling

## Abstract

This study investigates the influence expression of the MYCN oncogene has on the DNA damage response, replication fork progression and sensitivity to PARP inhibition in neuroblastoma. In a panel of neuroblastoma cell lines, *MYCN* amplification or MYCN expression resulted in increased cell death in response to a range of PARP inhibitors (niraparib, veliparib, talazoparib and olaparib) compared to the response seen in non-expressing/amplified cells. MYCN expression slowed replication fork speed and increased replication fork stalling, an effect that was amplified by PARP inhibition or PARP1 depletion. Increased DNA damage seen was specifically induced in S-phase cells. Importantly, PARP inhibition caused a significant increase in the survival of mice bearing MYCN expressing tumours in a transgenic murine model of MYCN expressing neuroblastoma. Olaparib also sensitized MYCN expressing cells to camptothecin- and temozolomide-induced cell death to a greater degree than non-expressing cells. In summary, MYCN expression leads to increased replication stress in neuroblastoma cells. This effect is exaggerated by inhibition of PARP, resulting in S-phase specific DNA damage and ultimately increased tumour cell death. PARP inhibition alone or in combination with classical chemotherapeutics is therefore a potential therapeutic strategy for neuroblastoma and may be more effective in MYCN expressing tumours.

## INTRODUCTION

Neuroblastoma (NB), a tumour derived from primitive neural crest cells in the sympathetic nervous system, is the most common extracranial solid childhood tumour [1, 2]. Patients with NB are stratified into risk groups depending on a number of features, including age at diagnosis, stage, *MYCN* amplification status and DNA ploidy [3]. At the time of diagnosis, the majority of patients have high-risk disease, defined as the presence of stage IV disease or amplification of the *MYCN* oncogene. *MYCN* amplification is present in 25% of NB patients and strongly predicts poor prognosis independently of other factors [4, 5]. The majority of patients with amplification also display high MYCN expression. With recent intensification of treatment, survival in *MYCN* amplified patients has improved so that outcomes are now comparable with other high-risk patients. However, approximately half of children with high-risk NB still relapse and die of their disease despite intensive therapies including multi-agent induction chemotherapy, surgery, radiotherapy, high-dose chemotherapy with autologous stem cell transplant, differentiation therapy and anti GD2 immunotherapy. We are now at the stage where conventional therapy is at the limits of tolerability and hence novel therapies targeting the molecular drivers of NB are urgently needed. As a driver of neuroblastoma, associated with poor outcome, MYCN is an important potential therapeutic target for high-risk NB. Whilst it seems intuitive to target MYCN directly, this has proved technically difficult [6]. Increased understanding of MYCN biology is needed in order that alternative ways to exploit MYCN expression can be explored.

Poly(ADP-ribose) polymerase (PARP) enzymes PARP1, PARP2 and PARP3 bind to, and are activated at, sites of DNA damage. Here they synthesise poly(ADP-ribose) (PAR) chains on acceptor proteins as well as themselves [7, 8]. The PAR signal then recruits repair factors to the damage, including PARP proteins that play a key role in coordinating the repair of single strand [9–16] and double strand DNA breaks [17–20] and in the restart of stalled or collapsed DNA replication forks [21–23]. PARP inhibitors, targeting PARPs 1, 2 and 3 to various degrees, are considered an exciting prospect for treatment of cancers with particular genetic alterations [24]. Several are approved for use in BRCA-defective high-grade serous ovarian cancer and in BRCA1/2 mutant HER2 negative breast cancers, while multiple trials in other homologous recombination deficient tumour types are still ongoing. In addition, PARP inhibitors effectively sensitize tumour cells to other DNA damaging agents. Recently it has been shown that NB cells with MYCN expression have higher levels of PARP1/2 and that at relatively high concentrations the PARP inhibitor olaparib can selectively kill NB cell lines expressing MYCN [25]. It is purported that this is because PARP inhibitors induce high levels of replication stress in MYCN expressing tumours. However, other reports do not confirm sensitivity to PARP inhibitors despite seeing significant alterations in levels of replication stress [26, 27].

Here we show directly that expression of the oncogene MYCN induces collapse of replication forks and sensitivity to the PARP inhibitors olaparib, niraparib and veliparib in a number of *MYCN*-amplified and expressing NB tumour cell lines, causing replication stress and increased DNA damage in S and G_2_/M cells. Significantly, we also demonstrate for the first time that treatment with olaparib (a PARP1/2/3 inhibitor) moderately increases survival in a transgenic murine model of MYCN expressing NB. Our work provides mechanistic insight into the relationship between PARP and MYCN and adds to the growing evidence that PARP inhibitors may have therapeutic potential in the treatment of MYCN expressing NB.

## RESULTS

### MYCN expression increases the sensitivity of NB cells to PARP inhibition

**Figure 1:**
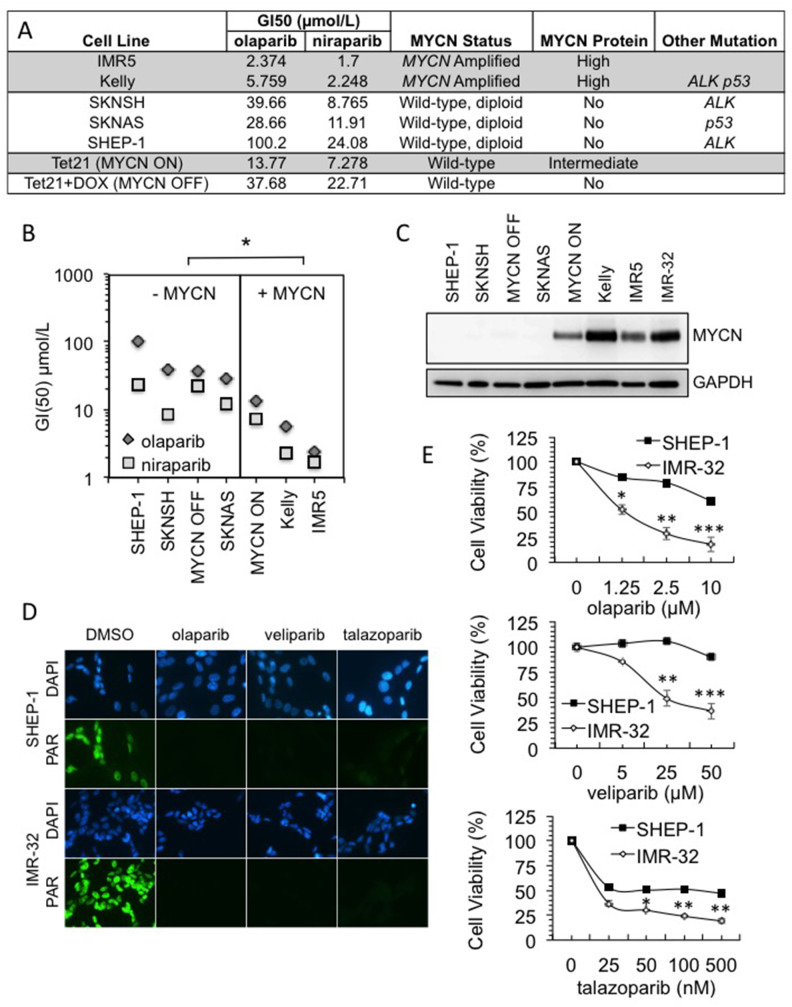
*MYCN* amplification influences sensitivity to PARP inhibition in a range of NB cell lines **(A)** GI50s of PARP inhibitors olaparib and niraparib in NB cell lines. *MYCN* gene status, MYCN expression status and other common mutation status are shown. Highlighting indicates the cell lines with MYCN expression. **(B)** GI50 values plotted against cell lines grouped by MYCN status. Significance was calculated using Student’s t-test comparing MYCN expressing to non-expressing cell lines, for each PARP inhibitor: ^*^ = p<0.05. **(C)** Western blot showing MYCN expression in each of the cell lines used. **(D)** Olaparib, talazoparib and veliparib mediated inhibition of poly(ADP-ribose) (PAR) synthesis detected by immunofluorescence in IMR-32 and Shep-1 NB cell lines. Cells were pre-treated for 16 hours with 10 nM talazoparib, 1 μM olaparib or 1 μM veliparib, PARP activity was then induced with H_2_O_2_. Representative images are shown, PAR (green), DAPI (blue). **(E)** Cell viability determined by MTT assay after 96 hour treatment with: (i) olaparib; (ii) veliparib; and (iii) talazoparib in Shep-1 and IMR-32 NB cell lines. Statistical significance was calculated using the Student’s t-test, comparing Shep-1 to IMR-32 cells at each dose. In each case mean and SEM of 2 independent repeats each representing 8 replica data points are shown. ^*^,^**^,^***^ = p<0.05, p<0.01, and p<0.001, respectively.

Previous reports regarding the role of MYCN in the response to PARP inhibitors are contradictory [25–27]. Furthermore when MYCN dependent sensitivity has been seen it was reported as specific to PARP inhibitors that induce high levels of PARP trapping on DNA (i.e., olaparib and talazoparib but not veliparib after 48 h incubation [25]). Here, a panel of *MYCN*-amplified/expressing and non-amplified/expressing NB tumour cell lines were screened for reduced viability in the presence of the PARP inhibitors olaparib (AZD-2281) and niraparib (MK-4827) (Figure 1A and Supplementary Figure 1). The MYCN expressing cell lines (Kelly, IMR5, SHEP-Tet21N - MYCN ON) displayed significantly lower GI_50_ values than the non-expressing ones (SHEP-1, SKNSH, SHEP-Tet21N+DOX - MYCN OFF, SKNAS) (Figure 1B, Student’s t-test p<0.05 for both inhibitors). In addition, there appears to be a relationship between the degree of MYCN expression and sensitivity to PARP inhibition, with SHEP-Tet21/N cells having intermediate expression of MYCN and moderate GI_50_ values. These data therefore support a role for MYCN in influencing the response to PARP inhibition. Although the PARP inhibitor niraparib is reported as having slightly greater trapping ability than olaparib, both result in greater than five fold higher trapping than the PARP inhibitor veliparib (ABT-888) [28] and each has moderate trapping when compared to the second generation clinical PARP inhibitor talazoparib (BMN-673) [28, 29]. To examine further the ability of MYCN expression to influence sensitivity to PARP inhibition and to test the influence of trapping, the cytotoxicity of three PARP inhibitors, talazoparib, olaparib and veliparib, was compared by MTT assay in the NB cell lines, IMR-32 (*MYCN*-amplified) and SHEP-1 (non-*MYCN*-amplified). Inhibition of PARP activity was confirmed in each cell line using immunofluorescent staining (Figure 1D and Supplementary Figure 2). After 96 hours drug exposure, across a range of concentrations of each PARP inhibitor, the *MYCN*-amplified IMR-32 cells showed significantly reduced viability compared to non-amplified SHEP-1 cells (Figure 1E, Student’s t-test p<0.01 at the highest concentrations tested). This suggests that MYCN expression can influence response to a range of PARP inhibitors regardless of the degree of trapping. However, the potency of talazoparib was far greater in both *MYCN*-amplified and non-amplified cells. This could be due to differences in the IC50 values of each inhibitor [30–32], and/or because the degree of PARP trapping likely influences the sensitivity of NB cell lines to PARP inhibitors regardless of MYCN expression. Relative protein levels of MYCN in each cell line are shown in Figure 1C.

**Figure 2:**
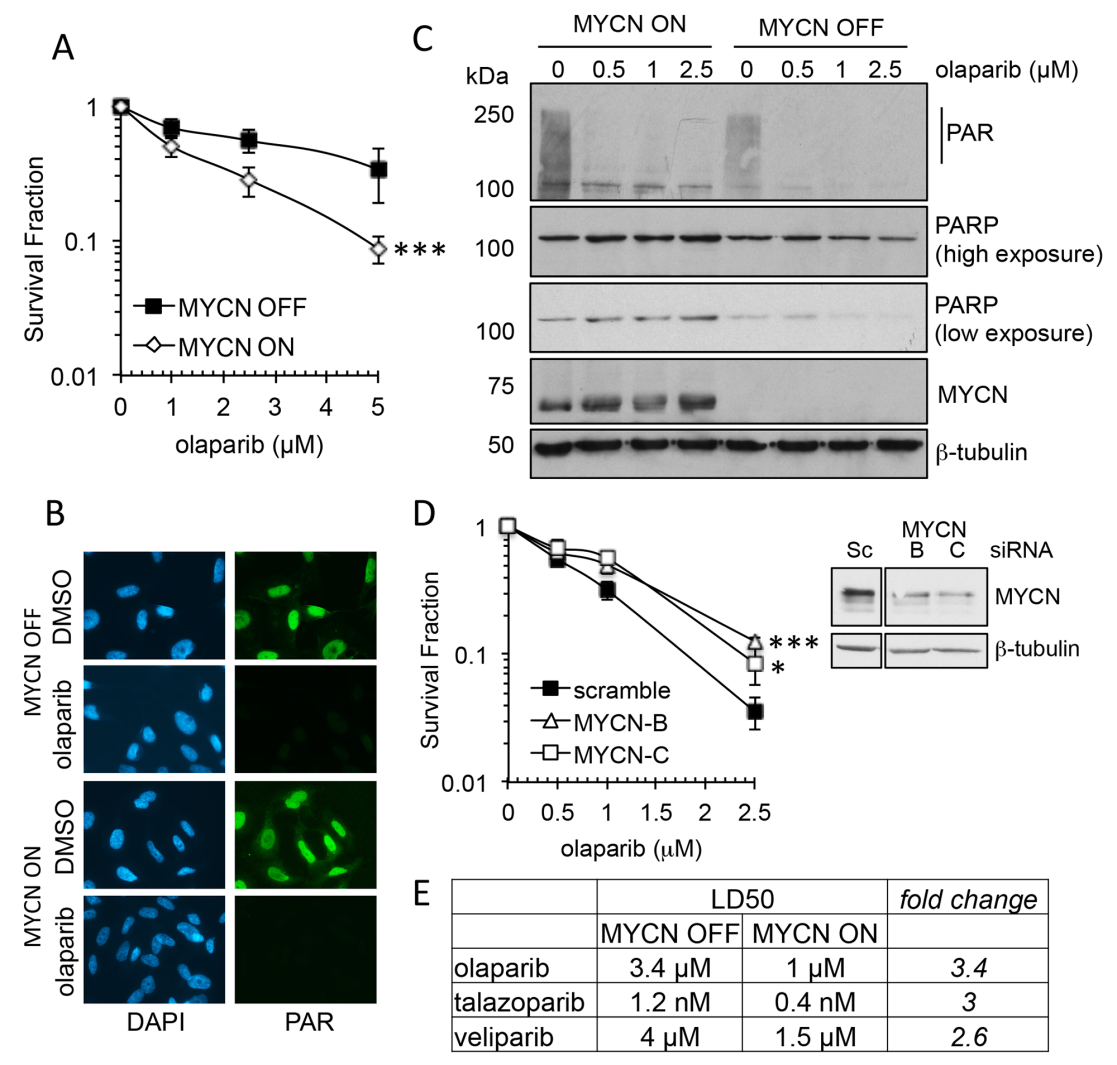
MYCN expression influences sensitivity to PARP inhibition in NB cell lines **(A)** Survival fraction of SHEP-Tet21/N NB cell line with MYCN ON or OFF as measured by clonogenic survival assay 14 days post treatment with olaparib. Statistical significance was calculated using the Student’s t-test, comparing MYCN OFF and MYCN ON cells. Mean and standard deviation of 3 independent repeats are shown. ^***^ = p<0.001. **(B)** Olaparib mediated inhibition of poly(ADP-ribose) (PAR) synthesis as detected by immunofluorescence in MYCN OFF and ON cell lines. Cells were pre-treated for 16 hours with 1 μM olaparib, PARP activity was then induced with H_2_O_2_. Representative images are shown, PAR (green), DAPI (blue). **(C)** Western Blot for PAR, PARP1, MYCN and β-actin, in MYCN ON and OFF cells 16 h post treatment with olaparib. **(D)** Survival fraction of IMR-32 cells transfected with MYCN targeting siRNA prior to treatment with olaparib measured by clonogenic survival assay. Mean and standard deviation of 3 independent repeats are shown. Statistical significance was calculated using the Student’s t-test, at 2.5 μM olaparib, where ^*^ = p<0.05, ^**^ = p<0.01. Protein expression of IMR-32 NB cells following siRNA transfection targeting MYCN is also shown. **(E)** LD_50_ values calculated from survival curves of SHEP-Tet21/N NB cell line with MYCN ON or OFF that were generated by clonogenic survival assay (above and Supplementary Figure 3) following exposure to the PARP inhibitors olaparib, talazoparib, and veliparib.

The MTT assay estimates cell viability. In order to assess cell survival following PARP inhibition clonogenic survival assays were undertaken, and to clarify the role of MYCN an isogenic model of the MYCN tet-repressible cell line, SHEP-Tet21/N was used [33]. MYCN expression was repressed for 48 h prior to continuous exposure to the PARP inhibitors olaparib, talazoparib or veliparib (Supplementary Figure 3). In this model MYCN expressing cells (MYCN ON) displayed significantly reduced cell survival compared to non-expressing (MYCN OFF) cells with each of the PARP inhibitors (Figure 2A and Supplementary Figure 3, Student’s t-test p<0.001 at 5 μM olaparib, p<0.01 at 5 nM talazoparib and p<0.05 at 5 μM veliparib). As with the cell viability assays, talazoparib was the most potent inhibitor regardless of MYCN expression, however, for each inhibitor the LD_50_ was approximately 3× higher in MYCN OFF than MYCN OFF cells (Figure 2E), suggesting that in this context trapping may not be the main factor driving cytotoxicity. The PARP inhibitor olaparib is used for the rest of this study. Adding tetracycline to the parental SHEP-1 cells did not affect cell survival in olaparib confirming the effect is due to expression of MYCN rather than off target effects of tetracycline (Supplementary Figure 4A). Inhibition of PARP activity was confirmed by immunofluorescent staining and western blotting and MYCN expression was confirmed by western blotting (Figure 2B and 2C and Supplementary Figure 5). To further demonstrate that MYCN can affect sensitivity to olaparib, MYCN was knocked down in *MYCN*-amplified IMR-32 cells using two different siRNAs and sensitivity to olaparib was tested by clonogenic survival (Figure 2D). Reduction of MYCN protein level significantly reduced sensitivity to olaparib with a 2.4–3.5 fold increase in cell survival at 2.5 μM (Student’s t-test, p<0.05 and <0.001 for MYCN siRNA-B and -C, respectively). Neither Ki67 staining or sensitivity to the cytotoxic agent etoposide were altered between MYCN depleted and control IMR-32 cells, demonstrating that the PARP inhibitor resistance induced is not simply a function of reduced proliferative fraction in the setting of reduced MYCN protein levels (Supplementary Figure 4B). Taken together these data strongly support the hypothesis that the presence of MYCN protein results in increased sensitivity to PARP inhibitors.

### MYCN expression is associated with increased PARP expression and activity in NB cells

**Figure 3:**
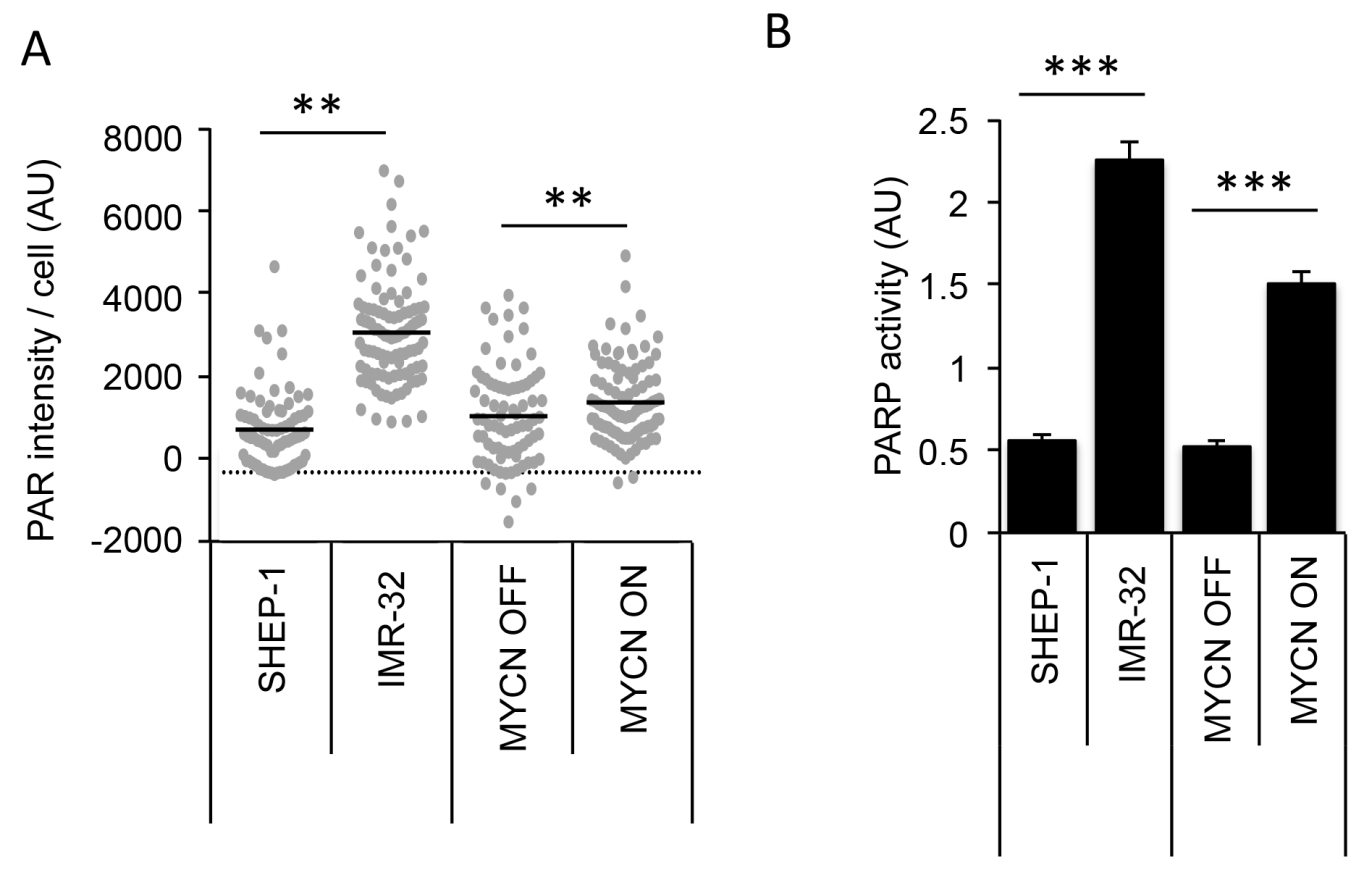
MYCN expression results in higher endogenous PARP activity in NB cell lines IMR-32 (*MYCN*-amplified), SHEP-1 (*MYCN* non-amplified) and SHEP-Tet21/N with MYCN expression ON or OFF were used. **(A)** Quantification of PAR immunofluorescence staining in NB cell lines. A minimum of 100 cells were analysed and statistical significance between cell lines was calculated using the Mann–Whitney U test where ^**^ represents p<0.01. **(B)** Quantification of PARP activity in NB cell lines as measured by the ability to add biotinylated PAR polymers onto histones, mean and standard deviation of triplicate repeats are shown. Statistical significance was calculated using the Student’s t-test, ^***^ = p<0.001.

Colicchia *et. al* demonstrated that PARP1 and PARP2 expression are significantly associated with high-risk NB and poor overall survival [25]. In addition, they demonstrated PARP protein levels are higher in MYCN expressing NB cell lines compared to non-expressing cells. Here, switching off MYCN in SHEP-Tet21/N cells was seen to slightly reduce expression of PARP1 protein and appeared to cause an associated reduction in endogenous PAR activity as indicated by western blotting (Figure 2C). In addition, the endogenous activity of PAR also differed when observed by immunofluorescent staining of PAR in MYCN ON and in *MYCN*-amplified IMR-32 cells (Figures 1 and 2, and quantified in Figure 3A, Mann–Whitney U test p<0.01 when comparing both IMR-32 with SHEP-1 and MYCN ON with MYCN OFF). Importantly, PARP activity was confirmed in *MYCN*-amplified IMR-32, non-amplified SHEP-1, MYCN OFF and MYCN ON cells using a highly sensitive *in vitro* quantitative assay (Figure 3B). Both *MYCN*-amplified and MYCN expressing cells displayed significantly higher levels of PARP activity than non-amplified/expressing cells (Student’s t-test p<0.001), confirming increased endogenous PARP activity is correlated to MYCN expression.

### Expression of MYCN and/or inhibition of PARP induces DNA damage in NB

**Figure 4:**
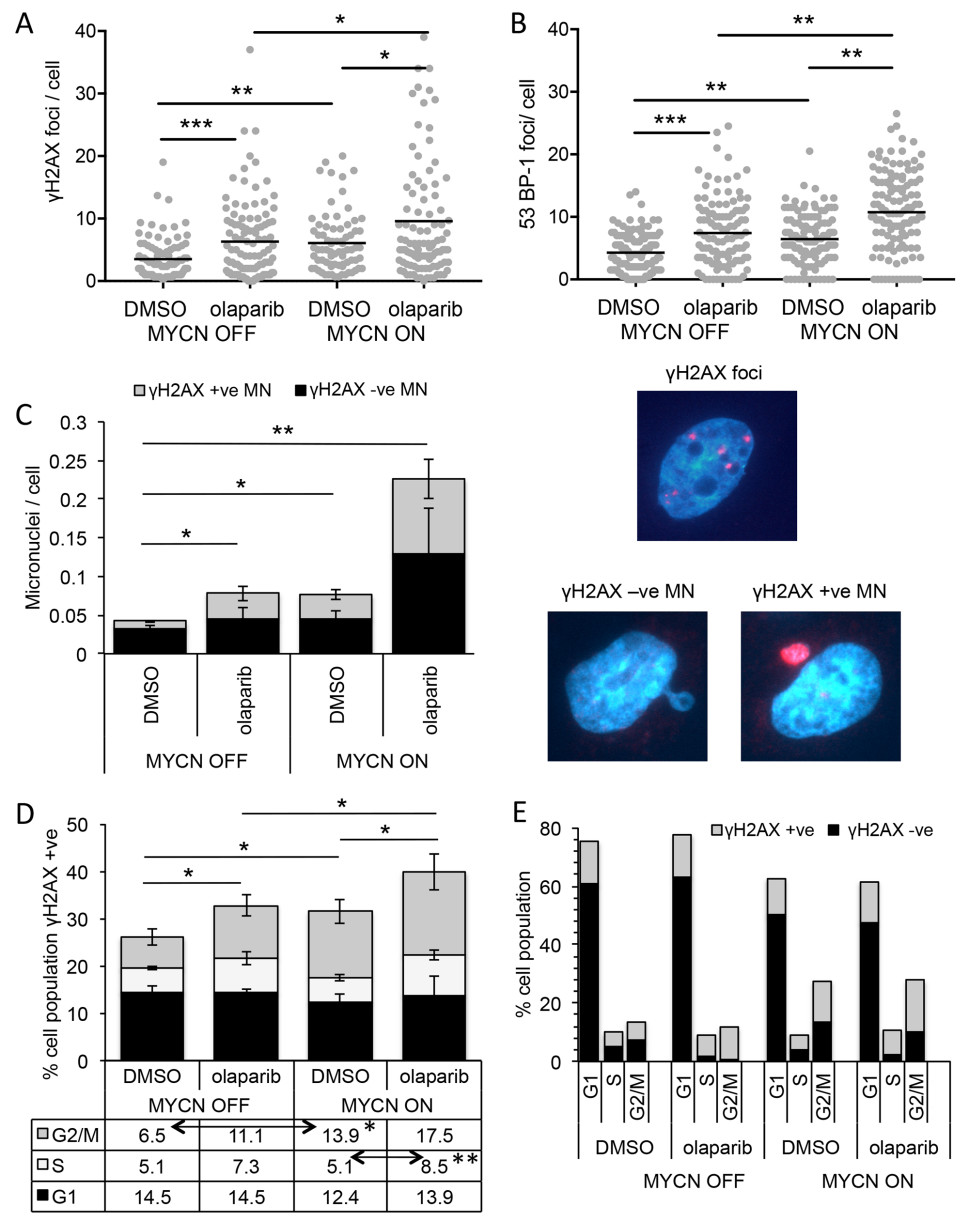
PARP inhibition induces more DNA damage and DNA double-strand breaks in NB cells when MYCN is expressed, predominantly in the S-phase of the cell cycle **(A)** Number of γH2AX and **(B)** 53BP-1 foci/cell in SHEP-Tet21/N cells with MYCN ON and OFF 16 hours post treatment with 1 μM olaparib or DMSO control. Data shown are pooled from three independent repeats, for each repeat n>50 cells. Statistical significance was calculated using the Mann–Whitney U test. **(C**, left**)** Number of γ-H2AX positive and negative micronuclei (MN)/cell in MYCN ON and MYCN OFF cells 16 hours post treatment with 1 μM olaparib or DMSO control. Mean +/− SEM of three independent repeats are shown, where >50 cells were counted on each occasion; significance indicated is between total MN/cell. Statistical significance was calculated using the Student’s t-test. RIGHT: examples of γ-H2AX foci and γ-H2AX +ve and −ve MN **(D)** Percentage of total cell population γH2AX positive in each phase of cell cycle and **(E)** cell cycle profile of all cells regardless of γH2AX, 10 hours post treatment of MYCN OFF and MYCN ON cells with 1 μM olaparib or DMSO control. Data in (D) and (E) are the mean and SEM of 3 independent repeats each representing 10,000 cells, significance above the graph compares total γH2AX positive under each condition, indicated significances in table below are between γH2AX positive in each phases of the cell cycle and were calculated by Student’s t-test. Throughout significance is indicated ^*^, ^**^, ^***^ = p<0.05, <0.01, and <0.001 respectively.

PARP plays multiple roles in repair of both double and single strand DNA breaks, and PARP inhibitors are generally considered to cause cell death by interfering with these functions. In order to examine the mechanism of PARP inhibitor mediated cell death in NB, levels of DNA damage were examined in the SHEP-Tet21/N MYCN ON and MYCN OFF cells in the presence and absence of the PARP inhibitor olaparib. Expression of MYCN alone resulted in a small but significantly increased level of DNA damage as visualised by immunofluorescent staining of γH2AX foci and 53BP-1 foci (Figure 4 and Supplementary Figure 6, γH2AX mean foci/cell MYCN OFF = 4.28 c.f MYCN ON = 6.44, Mann–Whitney U test p<0.01, 53-BP-1 mean foci/cell MYCN OFF = 3.51 c.f MYCN ON = 6.08, Mann–Whitney U test p<0.01). Inhibition of PARP increased the number of γH2AX foci/cell in both MYCN expressing and non-expressing cells consistent with increased levels of DNA damage, with an approximate 2-fold increase in mean foci/cell in each case (Figure 4A, Mann–Whitney U test p<0.05 and p<0.001, respectively). In addition, a similar increase in 53BP-1 foci was seen (Figure 4B, Mann–Whitney U test p<0.01 and p<0.001, respectively) indicative specifically of DNA double strand breaks. Together these data suggest that increased levels of DNA damage (most likely double strand breaks) may be the cause of olaparib induced cell death in NB, with higher cytotoxicity in MYCN expressing cells being the result of overall higher levels of DNA damage.

### PARP inhibitor induced DNA damage in NB cells expressing MYCN is associated with S phase of the cell cycle

During the examination of γH2AX foci, it was noted that PARP inhibition or expression of MYCN resulted in a small but significant increase in the number of micronuclei (MN)/cell (Student’s t-test p<0.05 in each case). Moreover, the additional MN were uniformly labelled with γH2AX (Figure 4C and Supplementary Figure 7). The combination of MYCN expression and PARP inhibition led to a much larger four fold increase in MN compared to MYCN OFF DMSO control (Student’s t-test p<0.01,), approximately half of which were γH2AX labelled. Such foci have been reported as associated with DNA replication stress [34, 35]. Further, PARP inhibition has been shown to increase DNA double strand breaks by inducing replication fork collapse during S-phase of the cell cycle [24]. In order to determine whether the increased DNA damage seen here is associated with any particular phase of the cell cycle, flow cytometry was used, co-staining for DNA with propidium iodide and DNA damage with a γH2AX antibody. In this assay, cells were classified as γH2AX foci positive or negative and the percentage of positive cells at each stage of the cell cycle determined (Figure 4D and Supplementary Figure 8); total cell cycle prolife (γH2AX positive plus and negative cells) is shown in Figure 4E. Consistent with γH2AX foci induction expression of MYCN or PARP inhibition significantly increased the total percentage of cells staining positive for γH2AX, with the largest γH2AX staining occurring in MYCN ON PARP inhibited cells (Student’s t-test p<0.05 in each case). Expression of MYCN resulted in an increase in the percentage of γH2AX positive cells, specifically in the G_2_/M phase of the cell cycle (Student’s t-test p<0.05, MYCN ON + DMSO c.f MYCN OFF + DMSO), likely reflective of the overall MYCN induced increase in G_2_/M phase cells. Addition of olaparib to MYCN OFF cells also resulted in an increase in percentage of γH2AX positive cells specifically in G_2_/M phase, although this was not statistically significant. No associated change in overall cell cycle profile was seen upon PARP inhibition. In MYCN ON cells, olaparib further increased the percentage of γH2AX positive cells. This increase was seen in S-phase (p<0.01, MYCN ON + DMSO c.f MYCN ON + olaparib) and G_2_/M phase cells, although again the increase in G_2_/M was not statistically significant. These data suggest that while MYCN or olaparib alone increase γH2AX in G_2_/M phase cells, only when they are combined can an increase in γH2AX in S-phase cells be detected.

### MYCN slows replication fork progression and induces replication fork stalling which is exacerbated by olaparib treatment

**Figure 5:**
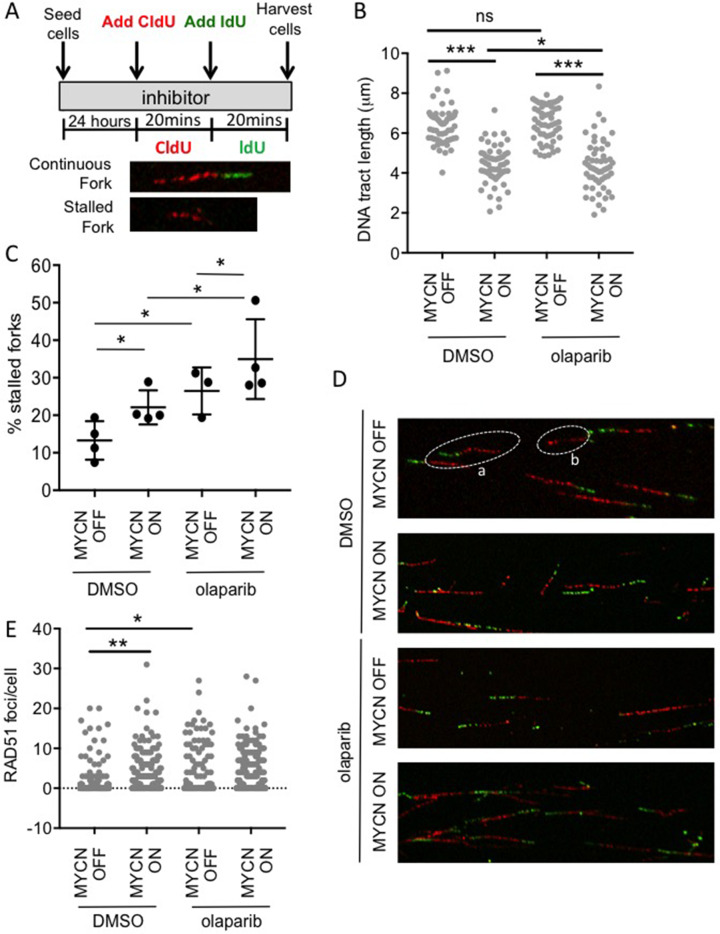
MYCN expression perturbs replication forks in NB cells and this is exacerbated upon PARP inhibition DNA fibre analysis of replication fork speed and stalling in olaparib treated SHEP-Tet21/N cells with MYCN ON and MYCN OFF cells. **(A)** Cells were incubated in 1 μM olaparib or DMSO control and then pulse labelled with CldU, for 20 min, and labelled switched to IdU for 20 min. **(B)** DNA fibre length (μm) and **(C)** Percentage fork stalling, calculated as a percentage (%) of CIdU only labelled tracts (red) from continuous forks (CIdU (red) and IdU (green) labelled tracts). For (B and C) at least 100 forks were counted on each of three separate occasions, (B) shows pooled data (means of individual repeats is shown in Supplementary Figure 9), (C) shows means of 3 independent repeats. Statistical significance was calculated using the Mann–Whitney U test (pooled data) and Student’s t-test (means). **(D)** Example images of replication forks, an example of continuous (a) and stalled forks (b) are circled. **(E)** Number of RAD51 foci/cell in MYCN ON or MYCN OFF cells 16 h post treatment with 1 μM olaparib or DMSO control. Data are pooled from three independent repeats for each repeat n>50 cells. Statistical significance was calculated using the Mann–Whitney U test. Throughout ^*^,^**^, and ^***^ represent p<0.05, <0.01, and <0.001, respectively.

It has previously been suggested that MYCN induces replication stress and that this makes cells sensitive to inhibition of PARP [25, 26, 36, 37]. Here, the progression of single replication forks was directly examined using the DNA fibre assay (Figure 5A-D & Supplementary Figure 9). In this assay, expression of MYCN significantly reduced the mean replication fork speed by 25% (Mann–Whitney U p<0.001), and increased replication fork stalling 1.7-fold, (Student’s t-test p<0.05). This is the first direct evidence that expression of MYCN causes replication fork stress in NB cells.

PARP1 is also reported as having a role at stalled replication forks where it both protects transiently stalled forks from collapse [38] and mediates MRE11-dependent replication restart and homologous recombination at collapsed forks [23]. Consistent with this function, exposure to olaparib significantly increased stalling of replication forks regardless of MYCN status. The overall level of stalled forks was highest in MYCN-expressing PARP inhibited cells, with 36% of replication forks stalled in MYCN ON olaparib treated cells. Mean fork speeds were also reduced by olaparib in both MYCN ON and OFF cells. Interestingly, this was only statistically significant in MYCN ON cells (Mann–Whitney U p<0.05), suggesting that although olaparib is having similar effects on fork progression in the presence or absence of MYCN, the overall effect of PARP inhibition on replication is greater when NB cells express MYCN. Depletion of PARP1 in MYCN ON and OFF NB cells also increased fork stalling, further supporting a protective function for PARP during NB replication (Supplementary Figure 9).

Perturbed replication forks can be restarted by two independent RAD51 pathways dependent on the nature of the lesion induced [39, 40]. Examination of RAD51 foci revealed that MYCN expression significantly increased RAD51 foci formation (Figure 5E and Supplementary Figure 10, p<0.01). In MYCN ON cells olaparib did not increase this level further, suggesting that whilst DNA damage is increased in MYCN expressing PARP inhibited cells, there is no corresponding increase in RAD51 mediated pathways for restart. It is likely that the overall effect on replication is sufficient to result in persistent DNA damage and therefore the increased PARP inhibitor induced toxicity seen in MYCN-amplified/expressing cells.

### Increased levels of DNA damage and replication fork stalling are also seen in MYCN amplified cells upon inhibition of PARP

Increased PARP inhibitor induced cell killing was seen in both MYCN ON v.s MYCN OFF cell lines and in a panel of MYCN amplified cell lines compared to non-amplified cells (Figure 1). Examination of γH2AX foci and DNA fibre analysis demonstrated that levels of DNA damage and replication fork stalling are also increased by PARP inhibition in the MYCN amplified neuroblastoma cell line IMR-32 (Supplementary Figure 11), suggesting that MYCN expression due to amplification also results in cell death due to effects on DNA fork stability and confirming that the findings in the MYCN ON/OFF expression system are conserved in MYCN amplified cells.

### Olaparib inhibits growth of MYCN expressing tumours *in vivo*

**Figure 6:**
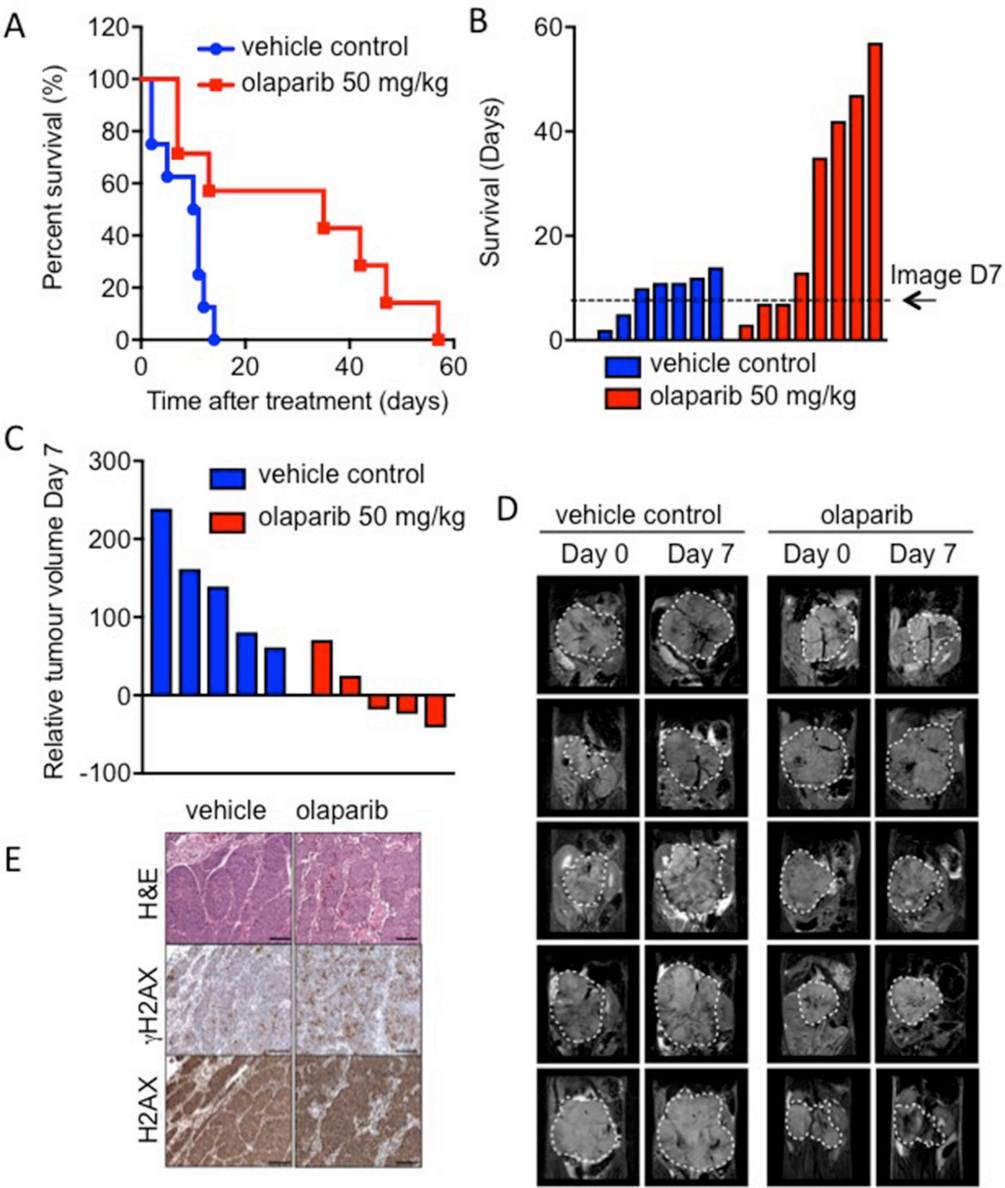
Olaparib significantly prolongs survival in the Th-*MYCN* mouse model of NB **(A)** Kaplan-Meier survival analysis for Th-*MYCN* animals (9 in vehicle control group, 8 in treatment group) treated daily with 50 mg/kg olaparib as a single agent. Treated group versus control group, p = 0.0186 by log-rank test. **(B)** Survival data as above but presented in days where each bar represents an individual animal. **(C)** Waterfall plot documenting relative changes in tumour volume in the Th-*MYCN* mouse model following seven-day treatment with daily dose of 50 mg/kg olaparib, p = 0.0159 (Mann–Whitney U test with a 5% level of significance). **(D)** Representative day 0 and 7 T_2_-weighted MRI abdominal sections of Th-*MYCN* mice treated daily with vehicle or with 50 mg/kg olaparib. Dashed white lines indicates tumour circumference. **(E)** Images of H&E and immunohistochemical staining for H2AX and γH2AX of Th-*MYCN* tumours.

**Figure 7:**
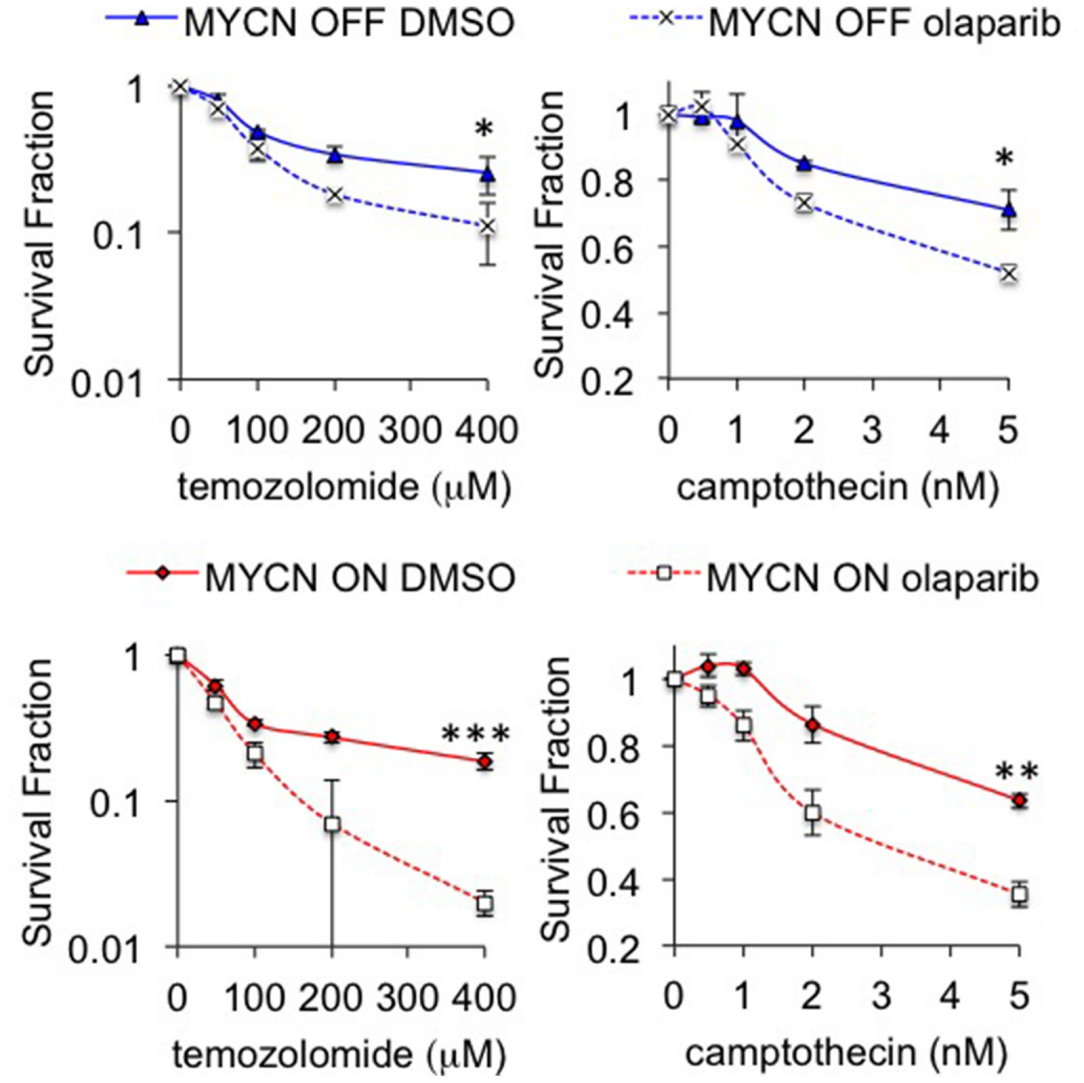
Inhibition of PARP sensitizes NB cells to camptothecin and temozolomide Cell viability determined by MTT assay after 96 hours treatment with 0.5 μM olaparib and temozolomide (left) or camptothecin (right) in MYCN OFF (top) and MYCN ON (bottom) cells. Statistical significance was calculated using the Student’s t-test, comparing with and without olaparib at the highest dose used. In each case mean and SEM of 2 independent repeats each representing 8 replica data points are shown. ^*^,^**^,^***^ = p<0.05, p<0.01, and p<0.001, respectively.

Despite promising *in vitro* data, a previous *in vivo* study of PARP inhibition in *MYCN* amplified NB failed to demonstrate any significant benefit on tumour growth or survival [27]. To further validate whether olaparib treatment is feasible in the context of MYCN expression, olaparib trials were conducted in transgenic Th-*MYCN* mice [41]. Mice bearing MYCN expressing tumours were treated with 50 mg/kg olaparib once daily and the long-term survival was assessed over the course of 8 weeks. Treatment with olaparib significantly prolonged the survival of animals (n = 8) compared to vehicle-treated animals (n = 9) (Figure 6A&B, log-rank p<0.05). Of the eight mice in the treatment group survival ranged from 7 to 57 days, while the control group range was 2–14 days. T_2_-weighted anatomical MRI demonstrated a marked tumour growth inhibition compared to vehicle controls (Figure 6C–6D (Supplementary Figure 12), Student’s t-test p<0.01). This is considered a moderate and optimistic response compared to NB standard therapies tested in the same model [42]. Increased levels of γH2AX staining in tumours from olaparib treated compared to control animals support of the idea that olaparib is reducing tumour death through increased levels of unresolved DNA damage (Figure 6E). These data support the potential of PARP inhibition in MYCN expressing tumours. Finally the ability of PARP inhibition to sensitize NB cells to the chemotherapeutic agents temozolomide and camptothecin was tested (Figure 7). Olaparib was able to sensitize both MYCN expressing and non-expressing cells to both agents suggesting that combination therapies may be clinically beneficial in NB patients.

## DISCUSSION

Our data support an emerging paradigm in which MYCN expression generates high levels of oncogenic replication stress in NB cells and that this can be exploited for therapeutic gain by inhibiting PARP.

It is well established that the Myc family of proteins induce replication stress and DNA damage leading to activation of the DDR [25, 43]. Utilising a DNA fibre assay we provide the first direct evidence of MYCN expression increasing replication stress in NB cells. MYCN expression resulted in slowed DNA replication and an increase in stalled replication forks. Fork stalling can be due to altered fork progression or changes in fork stability. Given that Myc proteins are thought to promote DNA replication it seems likely that MYCN alters fork progression either by promoting the cell cycle progression, by promoting transcription of related metabolic pathways, and/or by directly regulating replication origin firing. The induction of RAD51 foci in the presence of MYCN could suggest the presence of uncoupled, remodelled replication forks occurring at sites of replication stress [44]. Alternatively given the concurrent increase in DNA double strand breaks seen, it is possible that the stalled forks represent sites of collapsed replication forks. Such collapsed replication forks are normally repaired by homologous recombination [23] and this may also account for the increased RAD51 foci seen in MYCN expressing cells.

PARP1 and PARP2 have a complex relationship with DNA replication fork stalling/collapse. Both proteins are involved in single strand break repair (SSBR) [12], a lack of which can lead to increased collapsed replication forks [40]. In addition, they are themselves activated during replication stress. At hydroxyurea-induced transiently stalled forks, PARP1 serves to protect the fork from MRE11 mediated degradation [38, 39], while after prolonged stress with hydroxyurea, activated PARP1/2 mediates effective restart of the stalled fork via promotion of homologous recombination [23]. Furthermore during various mild replication stresses PARP1 has been shown to play a role in regulation of replication restart at reversed replication forks [44]. Regardless of MYCN status, it seems probable both inhibition of SSBR and destabilisation of collapsed, stalled or reversed forks contribute to the increased levels of DNA damage seen after olaparib treatment. However, when MYCN is expressed and replication stress increased, PARP inhibition is likely to have a greater effect due to lack of repair and/or protection of MYCN-induced perturbed forks. We therefore propose that when MYCN is expressed and PARP is inhibited, fork instability and DNA damage reach a critical point and cell death is induced. This explains increased sensitivity to PARP inhibition in NB cells expressing MYCN. The hypothesis is supported by the large number of replication associated MN and increase in S-phase associated γH2AX foci seen in olaparib treated MYCN expressing cells.

Interestingly, whilst RAD51 foci were increased in NB cells expressing MYCN, a further increase was not observed with olaparib treatment. Previous work has suggested that both remodelled fork restart during replication stress [44] and homologous recombination at stalled forks is reliant on PARP1 and PARP2 [23] and our data suggests this may be the case with MYCN-induced replication stress. However, the precise relationship between PARP, MYCN and replication fork dynamics requires further investigation.

Given its function as a transcription factor it seems probable that MYCN expression results in increased expression of DNA repair proteins to enable NB cells to cope with this replication stress. mRNA expression of the PARP family of proteins has been correlated with *MYCN* amplification prognosis in neuroblastoma [25] and here we provide *in vitro* evidence that PARP1 protein and PAR activity is increased in association with MYCN expression. In order to demonstrate a role for other key DDR proteins in *MYCN*-amplified NB gene expression data from a published RNAseq [45] cohort was interrogated (498 human NB samples). Increased expression of ATR, Chk1, Chk2, RAD51, BRCA1 and BRCA2 all predict a worse prognosis in NB and are significantly higher in cases with *MYCN* amplification (Supplementary Figure 13). A transcriptional role for MYCN in upregulating the DDR has been given further credence by the recent identification of MYCN binding sites in the promoters of PARP1, PARP2, BRCA1, RMI2, and TOPBP1, albeit in castration resistant prostate cancer [46].

Recent reports have suggested that another subset of high-risk neuroblastoma with 11q deletion may also be sensitive to PARP inhibition. It has been suggested that this is probably due to a deficiency in HRR associated genes *ATM*, *MRE11A, H2AFX*, and *CHEK1* [47–49]; such tumours very rarely have concurrent *MYCN* amplification. This raises the intriguing possibility that PARP inhibition may be effective in the two most common sub-groups of NB but for different reasons. In tumours with 11q deletion it is a specific defect in the DDR that renders sensitivity to PARP inhibition, exploiting the concept of synthetic lethality. In MYCN expressing disease it is likely a general over-reliance on the DDR due to oncogenic replication stress that leads to PARP inhibitor sensitivity.

Previous work has failed to reach a consensus concerning the potential of PARP inhibitors for use in NB [25–27] and only one recent report has specifically examined the effect of MYCN expression on efficacy and the association between replication stress and PARP inhibition [25]. Here, we demonstrate the efficacy of a range of PARP inhibitors in a number of NB cell lines and show this efficacy is related to MYCN expression. Further, we report for the first time that olaparib can be utilised to increase survival in a transgenic murine model of MYCN expressing NB. This model shows a moderate and optimistic response to monotherapy with the PARP inhibitor olaparib, although the effect is not comparable to standard therapies tested in the same model [42].

The majority of our data indicate that both MYCN expression and *MYCN* amplification are associated with PARP inhibitor sensitivity. *MYCN* amplification leads to the overexpression of MYCN at both the mRNA and protein levels [50] and ectopic expression of MYCN is often used in pre-clinical models of high-risk disease [33, 51]. However, the clinical significance of MYCN expression without *MYCN* amplification is uncertain, with some reports suggesting it confers an unfavourable prognosis [52] whilst paradoxically others suggest it is associated with better outcomes [53]. We evaluated the efficacy of olaparib in the TH-*MYCN* transgenic mouse model, which leads to high MYCN expression specifically in neural crest lineage cells, and is a widely used genetic model of high-risk neuroblastoma [54]. However, given MYCN expression is driven by the TH promoter rather than by genomic amplification it is not a true model of *MYCN* amplified neuroblastoma. Interestingly, a previous *in vivo* study used a *MYCN*-amplified cell line in a xenograft model and found a PARP inhibitor did not affect tumour growth [27]. Another limitation of our study is that we have not evaluated PARP inhibitors in cell line models of progressive disease, which have undergone intensive prior therapy exposure. Early phase clinical trials are likely to be heavily reliant on such cases. We therefore suggest further studies with additional *in vitro* and *in vivo* models of high-risk and progressive neuroblastoma are required before clinical testing of PARP inhibitors in children with neuroblastoma. It may also be that PARP inhibitors are more effective in combination with other DNA damaging agents, indeed our initial investigations show that olaparib can sensitize to both temozolomide and camptothecin, with a greater fold increase in sensitization being seen in MYCN expressing cells (Figure 7). The S-phase specific interaction between MYCN and PARP is likely to guide which agents will give the largest effects in high-risk NB, and will be the focus of future research.

In conclusion, we show that MYCN induces replication stress in NB cells and that this is exacerbated with pharmacological PARP inhibition leading to the selective killing of NB cells expressing MYCN. We also confirm the *in-vivo* feasibility of using olaparib in NB using a transgenic model of MYCN expressing NB. Together with other work showing the effectiveness of PARP inhibitors in NB tumours with 11q deletion, our findings provide evidence for a potential role for PARP inhibitors in the management of high-risk NB.

## MATERIALS AND METHODS

### Cell culture

Kelly, SH-SY5Y, SHEP-1, SK-N-SH, IMR-32, SKN-Be2C, IMR5 and SK-N-AS human neuroblastoma cell lines were obtained from the University of California at San Francisco Cell Culture Facility (San Francisco, CA, USA) and from the American Type Culture Collection (Manassas, VA, USA) and were routinely authenticated by STR-analysis (Institute of Cancer Research, Sutton, UK). Cell lines were used between 2 and 20 passages of original stocks. MYCN status and PARP activity was confirmed before and during the experiments. SHEP-Tet21/N cells were a gift from Dr Deborah Tweddle (University of Newcastle, UK). SHEP-Tet21/N, SHEP-1 and IMR-32 cells were also STR tested retrospectively at the end of the study by Culture Collections, Public Health England, Porton Down, UK. Cell lines were routinely tested for mycoplasma by PCR (Sigma MP0035 LookOut^®^ Mycoplasma PCR Detection Kit). All cell lines were grown at 37°C in an atmosphere containing 5% CO_2_. IMR-32 cells were cultured in Dulbecco’s modified Eagle Medium (DMEM, Gibco, ThermoFisher Scientific, MA, USA) and RPMI-1640 Medium (Gibco, ThermoFisher Scientific, MA, USA) mixed in a 50:50 ratio and supplemented with 10% Foetal bovine serum (FBS, Gibco, ThermoFisher Scientific, MA, USA). SHEPTet21/N cells were grown in RPMI-1640 Medium supplemented with 10% tetracycline-free FBS (BioSera, UK). The SHEP-Tet21/N MYCN expression system was used as previously described to conditionally express MYCN in a non-*MYCN*-amplified background. MYCN expression was switched off by the addition of 1 μg/mL tetracycline 48 h prior experiments. All other cell lines were grown in DMEM supplemented with 10% FBS.

### PARP Inhibitors

Olaparib was purchased from Cambridge Biosciences (UK) and veliparib, talazoparib and niraparib (MK4827) were purchased from Selleckchem (UK). All were dissolved in 100% DMSO to give a 10 mM stock. When confirming inhibition of PARP, PARP inhibitors were added overnight (16 h) for convenience. Temozolomide and camptothecin were purchased from Sigma-Aldrich (UK).

### siRNA

IMR-32 cells were transfected with two unique 27-mer siRNA duplexes against human *MYCN* (siMYCNA, ACGCUGAUACAUAACUAAAUUUGAA; siMYCNB, 5-AGUUCAUACCUAAGUACUGUAAUAA-3; siMYCNC, 5-AGCUGAUCCUCAAACGAUGCCUUCC-3.) and the control duplex (Trilencer-27 Universal Scrambled Negative Control siRNA Duplex) (Origene, UK). Cells were transfected with 20 nM siRNA (final concentration) using Lipofectamine RNAiMAX transfection reagent (Life Technologies, CA, USA) in accordance with the manufacturer’s instructions.

### Calculation of GI_50_

3-(4,5-dimethylthiazol-2-yl)-5-(3-carboxy-metho xyphenyl)-2-(4-sulfophenyl)-2H-tetrazolium (MTS) assay (Promega, WI, USA) was performed according to the manufacturer’s instructions. Cells were seeded in 96-well plates 24 hr before treatment. Stock solution of olaparib and niraparib was prepared in 100% DMSO, serially diluted in growth media to a range of concentrations and added to the cells resulting in a final volume of 200 μL per well. After 72 hr at 37°C, warmed MTS solution were added to each well and further incubated for 2 hr at 37°C before the absorbance was measured. GI50 was calculated using GraphPad Prism 5.0 (GraphPad software, CA, USA). Percentage survival was calculated as: % survival = (Abs treated − Abs blank)avg / (Abs control − Abs blank)avg × 100.

### MTT assay

Cells were plated in 96-well plates 24 hours prior to addition of inhibitors. After 96 h at 37°C MTT solution was added and plates incubated for 3 hours before formazan precipitate was dissolved DMSO and optical density read (Thermo Scientific Multiskan FC microplate reader). Cell viability was calculated compared to untreated controls. For sensitization assays temozolomide or camptothecin were added 4 hours after olaparib.

### Clonogenic survival assay

Cells were plated at known densities in 90 mm dishes. After 4 h PARP inhibitors were then added to the media. After 10–14 days colonies were stained with 4% methylene blue in 70% methanol and counted. In experiments using siRNA knockdown, cells were transfected in 6-well plates and left for 48 h before re-plating as above.

### Western blotting

Cells were lysed in RIPA buffer (50 mM Tris-HCl, 150 mM NaCl, 1% Triton X-100, 0.1% SDS, 1 mM EDTA, and 1% sodium deoxycholate) in the presence of 1× protease and phosphatase inhibitor cocktails (Sigma-Aldrich, MO, USA). Proteins were resolved by SDS-PAGE and transferred to Hybond ECL membrane (GE Healthcare, CO, USA). The membrane was immunoblotted with antibodies against Poly(ADP-ribose) 10H (ALX-804-220-R100, 1:400, Enzo Life Sciences, NY, USA), PARP1 (sc-8007, 1:1000, Santa Cruz Biotechnology, TX, USA), MYCN (sc-53993,1:250, Santa Cruz Biotechnology) and TUBB (T8328, β-tubulin; 1:5000, Sigma-Aldrich), each diluted in 5% milk and incubated at 4°C overnight. After the application of the appropriate HRP-conjugated secondary antibody and further washes, the immunoreactive protein was visualised using ECL reagents (GE Healthcare, IL, USA) according to the manufacturer’s instructions.

### Immunofluorescence

Cells were plated on to coverslips and allowed to adhere for 4 hours prior to treatment as indicated. Cells were fixed in 4% paraformaldehyde solution (Insight Biotechnology Ltd, UK) for 20 min at room temperature and extensively washed (3 × 5 min in tris-buffered saline (TBS), 1 × 10 min in phosphate-buffered saline (PBS) containing 0.5% Triton X-100 and 3 × 5 min in TBS). Coverslips were placed in 3% bovine serum albumin (BSA, Sigma-Aldrich, MO, USA), in TBS for 1 hour at room temperature to block followed by a further 3 × 5 min washes in TBS prior to incubation with the primary antibodies: anti-γH2AX (ser139) (#2577 Cell Signaling, MA, USA), RAD51 (sc-8349, Santa Cruz Biotechnology, TX, USA), Ki67 (ab15580, abcam, UK) or the PAR binding reagent MABE1016 (Millipore, MA, USA) (each diluted 1:500 in TBS containing 3% BSA for 16 hours at 4°C. The coverslips were subsequently washed 4 × 10 min in TBS followed by incubation with the secondary antibodies, Alexa-fluor 594 goat anti-rabbit IgG or Alexa-fluor 488 goat anti-Mouse IgG (ThermoFisher Scientific, MA, USA) diluted in TBS containing 3% BSA (1:500) for 1 hour at room temperature and finally washed 3 × 5 min TBS. The cells were washed 3 times in PBS with 1/1000 DAPI applied for the last wash. Finally, the coverslips were mounted on to microscope slides using Vectashield (Vector Laboratories, UK).

All images were obtained with a Zeiss LSM 510 inverted confocal microscope using a planapochromat 63×/NA 1.4 oil immersion objective and excitation wavelengths 488 nm, 546 nm, and 630 nm. Through focus maximum projection, images were acquired from optical sections 0.5 μM apart and with a section thickness of 1.0 μm. The frequency of cells containing foci was determined by counting at least 100 nuclei on each independent repeat. Images were processed for publication using the ImageJ NIH image processing software. The number of foci from at least 50 cells in each of three independent experiments was counted.

### Micronuclei scoring

Immunofluorescence for γH2AX was performed on cells as detailed above. Imaging was again performed using a Zeiss LSM 510 inverted confocal microscope and planapochromat 63×/NA 1.4 oil immersion objective. Micronuclei (MN) were first identified using DAPI staining. MN were then examined for the presence or absence of γ-H2AX signal. A MN was designated as MN-γ-H2AX (+) if it was uniformly stained with γ-H2AX. MN were scored as either negative or positive for γH2AX staining and the average number of micronuclei of either type was calculated from the total number of cells counted. Cells with three or more MN were not included to avoid possible artefacts due to catastrophic cellular events. MN from three independent experiments were counted.

### γ-H2AX and cell cycle analysis

Cells were seeded in 90 mm dishes and left to attach overnight prior to treatment as indicated. Cells were then fixed in 70% methanol and stored overnight at −20°C. After washing in PBS, cells were resuspended in 2mLs PBS supplemented with 0.5% BSA (Sigma-Aldrich) and 0.25% Triton-X100. Following 15 min incubation on ice, cells were resuspended with the antibody, anti-γH2AX [(ser139) (#2577 Cell Signaling, MA, USA), 1:50] diluted in 50 μL of PBS supplemented with 0.5% BSA and 0.25% Triton-X100 and incubated for 2 hours. Cells were then washed with 0.25% Triton-X100 in PBS and incubated with the secondary antibody Alexfluor 488 goat anti-rabbit IgG (1:500, diluted in 100 μL PBS supplemented with 1% BSA) for 30 min protected from light. Following a final wash with PBS, cells were incubated with 5 μL RNaseA (2 mg/mL) and 200 μL propidium iodide (PI, 50 μg/mL) for 15 minutes in the dark. Samples were analysed by flow cytometry using the FACSCalibur 488 nm laser (BD Biosciences, CA, USA) and more than 10000 cells were counted. All experiments were repeated three times. Data were analysed using FLOJO software (Tree Star, San Carlos, CA, USA).

### DNA fibre analysis

Cells were seeded in a six well plate and left to attach for at least four hours prior to olaparib treatment overnight. The next day, chlorodeoxyuridine (CldU, Sigma-Aldrich, MO, USA) was added to the media to a final concentration of 25 μM and the cells were incubated for 20 minutes. 5-iodo-2′-deoxyuridine (IdU, Sigma-Aldrich, MO, USA) was then added to media to a final concentration of 250 μM for 20 minutes before washing with PBS. Cells were collected using trypsin and resuspended in cold PBS to a final volume of 4 × 10^5^ cells/mL. 2 μL of cells were mixed with 7 μL of spreading buffer (200 mM Tris-HCl, pH 7.4, 50 mM EDTA, and 0.5% SDS) on a glass slide. After incubation for 2 minutes, the slides were tilted 15–45° to allow the DNA spreads to run down the slide taking 3–5 minutes to reach the bottom edge. The DNA spreads were then air dried, fixed in 3:1 methanol/acetic acid, and refrigerated overnight. The next day, the DNA fibres were denatured in 2.5 M HCl for 1 h, washed with PBS, and blocked with 1% BSA in PBS-T (PBS and 0.1% Tween 20) for 1 hour. The newly replicated CldU and IdU tracks were labelled for 1 hour with the primary antibodies (1:1000 rat anti-BrdU antibody [BU1/75 (ICR1)] (ab6326, abcam, UK) and 1:750 mouse α-BrdU (Clone: B44, #347580 BD Biosciences, UK). After rinsing with PBS 3 times, secondary antibodies were applied (α-rat AlexaFluor 555 and α-mouse AlexaFluor 488, both at 1:500). After further washing with PBS, coverslips were applied using Vectashield (Vector Laboratories, UK) and after drying slides were stored at −20°C. The DNA fibres were visualised using an Olympus FV1000 confocal microscope with a PLAPON 60× oil objective lens. Lasers of 488 and 542 nm wavelength were used to visualise AlexaFluor 488 and AlexaFluor 555, respectively. Analysis was performed using the ImageJ NIH image processing software.

### PARP enzyme assay

Endogenous PARP-1 enzyme activity in IMR-32, SHEP-1 and Sheptet21N cells with MYCN ON or OFF was determined using PARP/Apoptosis Colorimetric Assay Kit (Trevigen, MD, USA) following manufacturer’s instructions. In each case PARP activity was measured in 500 cells. The results are presented as Units PARP/500 cells in artificial units.

### *In vivo* trial with Th-MYCN mice

All experimental protocols were monitored and approved by The Institute of Cancer Research Animal Welfare and Ethical Review Body, in compliance with guidelines specified by the U.K. Home Office Animals (Scientific Procedures) Act 1986, the United Kingdom National Cancer Research Institute guidelines for the welfare of animals in cancer research [55], and the ARRIVE guidelines [56]. Transgenic Th-*MYCN* mice were genotyped to detect the presence of human *MYCN* transgene [41]. Mice were housed in specific pathogen-free rooms in autoclaved, aseptic microisolator cages with a maximum of 4 animals per cage. Mice were allowed access to sterile food and water *ad libitum*.

Th-*MYCN* mice were treated with olaparib. Olaparib (50 mg/mL in 100% DMSO) was solubilized and frozen at −20°C. On the day of treatment, the solution was thawed and diluted in 10% (w/v) 2-Hydroxypropyl)-β-cyclodextrin (HBC; Sigma, Ayrshire, UK) in PBS. Tumour development was monitored weekly by palpation by an experienced animal technician. Mice with palpable tumours were then allocated into 2 treatment groups: olaparib-treated and vehicle controls (8–9 mice per group), unequal numbers were regrettably due to the poor penetrance (~11%) and long latency (mice develop tumours at 55–160 days with a mean age of 79 days at the time of enrolment) in the TH-*MYCN* heterozygous animal, 9 animals were kept in the control group for sensitivity and precision. This number was determined to be high enough for statistical power. Olaparib was dosed by intraperitoneal (i.p.) injection once daily for 4 weeks. Tumour growth was monitored, as previously described [42], using T_2_-weighted magnetic resonance imaging (MRI) on a 7T MicroImaging system (Bruker Instruments, Ettlingen, Germany) performed on days 0 and 7. The mean tumours volume between the vehicle- and olaparib treated cohorts were not statistically different at the time of enrolment (1391 ± 212 mm^3^ vs 1167 ± 158 mm^3^, mean ± 1 s.e.m, p = 0.4) For mice that had to be sacrificed/died before/on day 7 and when imaging could not be performed, only survival data is presented. Individual group comparisons were performed using Student’s independent t-test and data were reported as significant where P<0.05.

Group sizes were determined by power analyses using data from previous publications. A cohort size of 8 mice was chosen for this study with >30% effect size, 5% significance level using non parametric test and a minimum power of 95%, (minimum size = 4). No randomization was done due to the poor penetration and long latency of the model. Mice were assigned to either vehicle control or treatment in turn when a tumour was identified by palpation

### Immunohistochemistry staining of fixed tissues

Animal tumours were harvested at sacrifice, fixed in 10% (v/v) neutral buffered formalin, and paraffin-embedded for histologic studies. Four μm sections of paraffin-embedded tumours were cut using a Leica microtome RM2235. The tissue sections were deparaffinised and rehydrated in water prior to antigen retrieval using 1% (v/v) citric acid for 23 min in a microwave (3 min at full power and additional 20 min at 40% of power), followed by a 25 min wash in 1% (w/v) hydrogen peroxide (Hydrogen peroxide 30% (w/v) aqueous solution AnalaR NORMAPUR^®^, VWR, Lutterworth, UK). VECTOR^®^ M.O.M.™ immunodetection kit BASIC (Vector Laboratories, Peterborough, UK) was used. Non-specific antibody reactivity was blocked by incubation with M.O.M. IgG block containing 0.1% (v/v) Triton X-100 (Sigma, Ayrshire, UK) for mouse- or rat-derived antibodies or with 10% (v/v) BSA containing 0.1% (v/v) Triton X-100 for all other species for 90 min at room temperature (RT). The sections were then incubated overnight at RT using the following antibodies (mouse-derived antibodies in M.O.M. diluents, rabbit antibodies in 0.1% BSA) and dilutions: γH2AX (1:500; (#2577, Cell Signaling Technology, MA, USA) and H2AX (1:100; #2572, Cell Signaling Technology, MA, USA).

Sections were incubated for 10 min at RT with biotinylated anti-mouse antibody (1:250 in M.O.M. diluent) or biotinylated anti-rabbit antibody (1:500 in 0.1% BSA) followed by NeutrAvidin peroxidase (1:1000 from 1 mg/ml stock) incubation for 2 hr at RT. Detection was performed using ImmPACT DAB (Vector Laboratories, Peterborough, UK). For negative controls, the primary antibody was replaced by BSA. Slides were counterstained with haematoxylin and mounted/coverslips with Histolab Pertex^®^ (Algol Diagnostics, Finland).

### Statistical analysis

Results were determined to be normally distributed or not using Shapiro-Wilk test for normality, prior to analysis with a paired Student’s t-test or a Mann–Whitney U test as relevant and indicated. p values below 0.05 were considered representative of data that were significantly different. Graphpad Prism 7 software was used for analysis of all data.

## SUPPLEMENTARY MATERIALS FIGURES



## References

[1] Brodeur GM . Neuroblastoma: biological insights into a clinical enigma. Nat Rev Cancer. 2003; 3:203–16. 10.1038/nrc1014. 12612655

[2] Maris JM . Recent advances in neuroblastoma. N Engl J Med. 2010; 362:2202–11. 10.1056/NEJMra0804577. 20558371 PMC3306838

[3] Cohn SL , Pearson AD , London WB , Monclair T , Ambros PF , Brodeur GM , Faldum A , Hero B , Iehara T , Machin D , Mosseri V , Simon T , Garaventa A , et al. and INRG Task Force. The International Neuroblastoma Risk Group (INRG) classification system: an INRG Task Force report. J Clin Oncol. 2009; 27:289–97. 10.1200/JCO.2008.16.6785. 19047291 PMC2650388

[4] Brodeur GM , Seeger RC , Schwab M , Varmus HE , Bishop JM . Amplification of N-myc in untreated human neuroblastomas correlates with advanced disease stage. Science. 1984; 224:1121–24. 10.1126/science.6719137. 6719137

[5] Seeger RC , Brodeur GM , Sather H , Dalton A , Siegel SE , Wong KY , Hammond D . Association of multiple copies of the N-myc oncogene with rapid progression of neuroblastomas. N Engl J Med. 1985; 313:1111–16. 10.1056/NEJM198510313131802. 4047115

[6] Whitfield JR , Beaulieu ME , Soucek L . Strategies to Inhibit Myc and Their Clinical Applicability. Front Cell Dev Biol. 2017; 5:10. 10.3389/fcell.2017.00010. 28280720 PMC5322154

[7] Schreiber V , Dantzer F , Ame JC , de Murcia G . Poly(ADP-ribose): novel functions for an old molecule. Nat Rev Mol Cell Biol. 2006; 7:517–28. 10.1038/nrm1963. 16829982

[8] Benjamin RC , Gill DM . Poly(ADP-ribose) synthesis in vitro programmed by damaged DNA. A comparison of DNA molecules containing different types of strand breaks. J Biol Chem. 1980; 255:10502–08. 6253477

[9] Ding R , Pommier Y , Kang VH , Smulson M . Depletion of poly(ADP-ribose) polymerase by antisense RNA expression results in a delay in DNA strand break rejoining. J Biol Chem. 1992; 267:12804–12. 1618781

[10] Trucco C , Oliver FJ , de Murcia G , Ménissier-de Murcia J . DNA repair defect in poly(ADP-ribose) polymerase-deficient cell lines. Nucleic Acids Res. 1998; 26:2644–49. 10.1093/nar/26.11.2644. 9592149 PMC147627

[11] Le Page F , Schreiber V , Dherin C , De Murcia G , Boiteux S . Poly(ADP-ribose) polymerase-1(PARP-1) is required in murine cell lines for base excision repair of oxidative DNA damage in the absence of DNA polymerase beta. J Biol Chem. 2003; 278:18471–77. 10.1074/jbc.M212905200. 12637553

[12] Fisher AE , Hochegger H , Takeda S , Caldecott KW . Poly(ADP-ribose) polymerase 1 accelerates single-strand break repair in concert with poly(ADP-ribose) glycohydrolase. Mol Cell Biol. 2007; 27:5597–605. 10.1128/MCB.02248-06. 17548475 PMC1952076

[13] Ström CE , Johansson F , Uhlén M , Szigyarto CA , Erixon K , Helleday T . Poly (ADP-ribose) polymerase (PARP) is not involved in base excision repair but PARP inhibition traps a single-strand intermediate. Nucleic Acids Res. 2011; 39:3166–75. 10.1093/nar/gkq1241. 21183466 PMC3082910

[14] El-Khamisy SF , Masutani M , Suzuki H , Caldecott KW . A requirement for PARP-1 for the assembly or stability of XRCC1 nuclear foci at sites of oxidative DNA damage. Nucleic Acids Res. 2003; 31:5526–33. 10.1093/nar/gkg761. 14500814 PMC206461

[15] Okano S , Lan L , Caldecott KW , Mori T , Yasui A . Spatial and temporal cellular responses to single-strand breaks in human cells. Mol Cell Biol. 2003; 23:3974–81. 10.1128/MCB.23.11.3974-3981.2003. 12748298 PMC155230

[16] Rulten SL , Cortes-Ledesma F , Guo L , Iles NJ , Caldecott KW . APLF (C2orf13) is a novel component of poly(ADP-ribose) signaling in mammalian cells. Mol Cell Biol. 2008; 28:4620–28. 10.1128/MCB.02243-07. 18474613 PMC2447129

[17] Morrison C , Smith GC , Stingl L , Jackson SP , Wagner EF , Wang ZQ . Genetic interaction between PARP and DNA-PK in V(D)J recombination and tumorigenesis. Nat Genet. 1997; 17:479–82. 10.1038/ng1297-479. 9398855

[18] Boulton S , Kyle S , Durkacz BW . Interactive effects of inhibitors of poly(ADP-ribose) polymerase and DNA-dependent protein kinase on cellular responses to DNA damage. Carcinogenesis. 1999; 20:199–203. 10.1093/carcin/20.2.199. 10069454

[19] Audebert M , Salles B , Calsou P . Involvement of poly(ADP-ribose) polymerase-1 and XRCC1/DNA ligase III in an alternative route for DNA double-strand breaks rejoining. J Biol Chem. 2004; 279:55117–26. 10.1074/jbc.M404524200. 15498778

[20] Rulten SL , Fisher AE , Robert I , Zuma MC , Rouleau M , Ju L , Poirier G , Reina-San-Martin B , Caldecott KW . PARP-3 and APLF function together to accelerate nonhomologous end-joining. Mol Cell. 2011; 41:33–45. 10.1016/j.molcel.2010.12.006. 21211721

[21] Yang YG , Cortes U , Patnaik S , Jasin M , Wang ZQ . Ablation of PARP-1 does not interfere with the repair of DNA double-strand breaks, but compromises the reactivation of stalled replication forks. Oncogene. 2004; 23:3872–82. 10.1038/sj.onc.1207491. 15021907

[22] Sugimura K , Takebayashi S , Taguchi H , Takeda S , Okumura K . PARP-1 ensures regulation of replication fork progression by homologous recombination on damaged DNA. J Cell Biol. 2008; 183:1203–12. 10.1083/jcb.200806068. 19103807 PMC2606964

[23] Bryant HE , Petermann E , Schultz N , Jemth AS , Loseva O , Issaeva N , Johansson F , Fernandez S , McGlynn P , Helleday T . PARP is activated at stalled forks to mediate Mre11-dependent replication restart and recombination. EMBO J. 2009; 28:2601–15. 10.1038/emboj.2009.206. 19629035 PMC2738702

[24] Bryant HE , Schultz N , Thomas HD , Parker KM , Flower D , Lopez E , Kyle S , Meuth M , Curtin NJ , Helleday T . Specific killing of BRCA2-deficient tumours with inhibitors of poly(ADP-ribose) polymerase. Nature. 2005; 434:913–17. 10.1038/nature03443. 15829966

[25] Colicchia V , Petroni M , Guarguaglini G , Sardina F , Sahún-Roncero M , Carbonari M , Ricci B , Heil C , Capalbo C , Belardinilli F , Coppa A , Peruzzi G , Screpanti I , et al. PARP inhibitors enhance replication stress and cause mitotic catastrophe in MYCN-dependent neuroblastoma. Oncogene. 2017; 36:4682–91. 10.1038/onc.2017.40. 28394338

[26] Petroni M , Sardina F , Heil C , Sahún-Roncero M , Colicchia V , Veschi V , Albini S , Fruci D , Ricci B , Soriani A , Di Marcotullio L , Screpanti I , Gulino A , Giannini G . The MRN complex is transcriptionally regulated by MYCN during neural cell proliferation to control replication stress. Cell Death Differ. 2016; 23:197–206. 10.1038/cdd.2015.81. 26068589 PMC4716299

[27] Norris RE , Adamson PC , Nguyen VT , Fox E . Preclinical evaluation of the PARP inhibitor, olaparib, in combination with cytotoxic chemotherapy in pediatric solid tumors. Pediatr Blood Cancer. 2014; 61:145–50. 10.1002/pbc.24697. 24038812 PMC3849815

[28] Murai J , Huang SY , Das BB , Renaud A , Zhang Y , Doroshow JH , Ji J , Takeda S , Pommier Y . Trapping of PARP1 and PARP2 by Clinical PARP Inhibitors. Cancer Res. 2012; 72:5588–99. 10.1158/0008-5472.CAN-12-2753. 23118055 PMC3528345

[29] Murai J , Huang SY , Renaud A , Zhang Y , Ji J , Takeda S , Morris J , Teicher B , Doroshow JH , Pommier Y . Stereospecific PARP trapping by BMN 673 and comparison with olaparib and rucaparib. Mol Cancer Ther. 2014; 13:433–43. 10.1158/1535-7163.MCT-13-0803. 24356813 PMC3946062

[30] Oplustil O’Connor L , Rulten SL , Cranston AN , Odedra R , Brown H , Jaspers JE , Jones L , Knights C , Evers B , Ting A , Bradbury RH , Pajic M , Rottenberg S , et al. The PARP Inhibitor AZD2461 Provides Insights into the Role of PARP3 Inhibition for Both Synthetic Lethality and Tolerability with Chemotherapy in Preclinical Models. Cancer Res. 2016; 76:6084–94. 10.1158/0008-5472.CAN-15-3240. 27550455

[31] Penning TD , Zhu GD , Gandhi VB , Gong J , Liu X , Shi Y , Klinghofer V , Johnson EF , Donawho CK , Frost DJ , Bontcheva-Diaz V , Bouska JJ , Osterling DJ , et al. Discovery of the Poly(ADP-ribose) polymerase (PARP) inhibitor 2-[(R)-2-methylpyrrolidin-2-yl]-1H-benzimidazole-4-carboxamide (ABT-888) for the treatment of cancer. J Med Chem. 2009; 52:514–23. 10.1021/jm801171j. 19143569

[32] Wang B , Chu D , Feng Y , Shen Y , Aoyagi-Scharber M , Post LE . Discovery and Characterization of (8S,9R)-5-Fluoro-8-(4-fluorophenyl)-9-(1-methyl-1H-1,2,4-triazol-5-yl)-2,7,8,9-tetrahydro-3H-pyrido[4,3,2-de]phthalazin-3-one (BMN 673, Talazoparib), a Novel, Highly Potent, and Orally Efficacious Poly(ADP-ribose) Polymerase-1/2 Inhibitor, as an Anticancer Agent. J Med Chem. 2016; 59:335–57. 10.1021/acs.jmedchem.5b01498. 26652717

[33] Lutz W , Stöhr M , Schürmann J , Wenzel A , Löhr A , Schwab M . Conditional expression of N-myc in human neuroblastoma cells increases expression of alpha-prothymosin and ornithine decarboxylase and accelerates progression into S-phase early after mitogenic stimulation of quiescent cells. Oncogene. 1996; 13:803–12. 8761302

[34] Xu B , Sun Z , Liu Z , Guo H , Liu Q , Jiang H , Zou Y , Gong Y , Tischfield JA , Shao C . Replication stress induces micronuclei comprising of aggregated DNA double-strand breaks. PLoS One. 2011; 6:e18618. 10.1371/journal.pone.0018618. 21525980 PMC3078113

[35] Gravells P , Neale J , Grant E , Nathubhai A , Smith KM , James DI , Bryant HE . Radiosensitization with an inhibitor of poly(ADP-ribose) glycohydrolase: A comparison with the PARP1/2/3 inhibitor olaparib. DNA Repair (Amst). 2018; 61:25–36. 10.1016/j.dnarep.2017.11.004. 29179156 PMC5765821

[36] Gu L , Chu P , Lingeman R , McDaniel H , Kechichian S , Hickey RJ , Liu Z , Yuan YC , Sandoval JA , Fields GB , Malkas LH . The Mechanism by Which MYCN Amplification Confers an Enhanced Sensitivity to a PCNA-Derived Cell Permeable Peptide in Neuroblastoma Cells. EBioMedicine. 2015; 2:1923–31. 10.1016/j.ebiom.2015.11.016. 26844271 PMC4703743

[37] Gu L , Smith S , Li C , Hickey RJ , Stark JM , Fields GB , Lang WH , Sandoval JA , Malkas LH . A PCNA-derived cell permeable peptide selectively inhibits neuroblastoma cell growth. PLoS One. 2014; 9:e94773. 10.1371/journal.pone.0094773. 24728180 PMC3984256

[38] Ying S , Hamdy FC , Helleday T . Mre11-dependent degradation of stalled DNA replication forks is prevented by BRCA2 and PARP1. Cancer Res. 2012; 72:2814–21. 10.1158/0008-5472.CAN-11-3417. 22447567

[39] Petermann E , Orta ML , Issaeva N , Schultz N , Helleday T . Hydroxyurea-stalled replication forks become progressively inactivated and require two different RAD51-mediated pathways for restart and repair. Mol Cell. 2010; 37:492–502. 10.1016/j.molcel.2010.01.021. 20188668 PMC2958316

[40] Saleh-Gohari N , Bryant HE , Schultz N , Parker KM , Cassel TN , Helleday T . Spontaneous homologous recombination is induced by collapsed replication forks that are caused by endogenous DNA single-strand breaks. Mol Cell Biol. 2005; 25:7158–69. 10.1128/MCB.25.16.7158-7169.2005. 16055725 PMC1190269

[41] Weiss WA , Aldape K , Mohapatra G , Feuerstein BG , Bishop JM . Targeted expression of MYCN causes neuroblastoma in transgenic mice. EMBO J. 1997; 16:2985–95. 10.1093/emboj/16.11.2985. 9214616 PMC1169917

[42] Jamin Y , Tucker ER , Poon E , Popov S , Vaughan L , Boult JK , Webber H , Hallsworth A , Baker LC , Jones C , Koh DM , Pearson AD , Chesler L , Robinson SP . Evaluation of clinically translatable MR imaging biomarkers of therapeutic response in the TH-MYCN transgenic mouse model of neuroblastoma. Radiology. 2013; 266:130–40. 10.1148/radiol.12120128. 23169794 PMC4298658

[43] Dominguez-Sola D , Ying CY , Grandori C , Ruggiero L , Chen B , Li M , Galloway DA , Gu W , Gautier J , Dalla-Favera R . Non-transcriptional control of DNA replication by c-Myc. Nature. 2007; 448:445–51. 10.1038/nature05953. 17597761

[44] Zellweger R , Dalcher D , Mutreja K , Berti M , Schmid JA , Herrador R , Vindigni A , Lopes M . Rad51-mediated replication fork reversal is a global response to genotoxic treatments in human cells. J Cell Biol. 2015; 208:563–79. 10.1083/jcb.201406099. 25733714 PMC4347635

[45] Su Z , Fang H , Hong H , Shi L , Zhang W , Zhang W , Zhang Y , Dong Z , Lancashire LJ , Bessarabova M , Yang X , Ning B , Gong B , et al. An investigation of biomarkers derived from legacy microarray data for their utility in the RNA-seq era. Genome Biol. 2014; 15:523–48. 10.1186/s13059-014-0523-y. 25633159 PMC4290828

[46] Zhang W , Liu B , Wu W , Li L , Broom BM , Basourakos SP , Korentzelos D , Luan Y , Wang J , Yang G , Park S , Azad AK , Cao X , et al. Targeting the MYCN-PARP-DNA damage response pathway in neuroendocrine prostate cancer. Clin Cancer Res. 2018; 24:696–707. 10.1158/1078-0432.CCR-17-1872. 29138344 PMC5823274

[47] Takagi M , Yoshida M , Nemoto Y , Tamaichi H , Tsuchida R , Seki M , Uryu K , Nishii R , Miyamoto S , Saito M , Hanada R , Kaneko H , Miyano S , et al. Loss of DNA Damage Response in Neuroblastoma and Utility of a PARP Inhibitor. J Natl Cancer Inst. 2017; 109:djx062. 10.1093/jnci/djx062. 29059438

[48] Carén H , Kryh H , Nethander M , Sjöberg RM , Träger C , Nilsson S , Abrahamsson J , Kogner P , Martinsson T . High-risk neuroblastoma tumors with 11q-deletion display a poor prognostic, chromosome instability phenotype with later onset. Proc Natl Acad Sci USA. 2010; 107:4323–28. 10.1073/pnas.0910684107. 20145112 PMC2840092

[49] Sanmartin E , Munoz L , Piqueras M , Sirerol JA , Berlanga P , Canete A , Castel V , Font de Mora J . Deletion of 11q in Neuroblastomas Drives Sensitivity to PARP Inhibition. Clin Cancer Res. 2017; 23:6875–87. 10.1158/1078-0432.CCR-17-0593. 28830922

[50] Slavc I , Ellenbogen R , Jung WH , Vawter GF , Kretschmar C , Grier H , Korf BR . myc gene amplification and expression in primary human neuroblastoma. Cancer Res. 1990; 50:1459–63. 2302711

[51] Olsen RR , Otero JH , García-López J , Wallace K , Finkelstein D , Rehg JE , Yin Z , Wang YD , Freeman KW . MYCN induces neuroblastoma in primary neural crest cells. Oncogene. 2017; 36:5075–82. 10.1038/onc.2017.128. 28459463 PMC5582212

[52] Chan HS , Gallie BL , DeBoer G , Haddad G , Ikegaki N , Dimitroulakos J , Yeger H , Ling V . MYCN protein expression as a predictor of neuroblastoma prognosis. Clin Cancer Res. 1997; 3:1699–706. 9815553

[53] Cohn SL , London WB , Huang D , Katzenstein HM , Salwen HR , Reinhart T , Madafiglio J , Marshall GM , Norris MD , Haber M . MYCN expression is not prognostic of adverse outcome in advanced-stage neuroblastoma with nonamplified MYCN. J Clin Oncol. 2000; 18:3604–13. 10.1200/JCO.2000.18.21.3604. 11054433

[54] Teitz T , Stanke JJ , Federico S , Bradley CL , Brennan R , Zhang J , Johnson MD , Sedlacik J , Inoue M , Zhang ZM , Frase S , Rehg JE , Hillenbrand CM , et al. Preclinical models for neuroblastoma: establishing a baseline for treatment. PLoS One. 2011; 6:e19133. 10.1371/journal.pone.0019133. 21559450 PMC3084749

[55] Workman P , Aboagye EO , Balkwill F , Balmain A , Bruder G , Chaplin DJ , Double JA , Everitt J , Farningham DA , Glennie MJ , Kelland LR , Robinson V , Stratford IJ , et al. and Committee of the National Cancer Research Institute. Guidelines for the welfare and use of animals in cancer research. Br J Cancer. 2010; 102:1555–77. 10.1038/sj.bjc.6605642. 20502460 PMC2883160

[56] Kilkenny C , Browne WJ , Cuthill IC , Emerson M , Altman DG . Improving bioscience research reporting: the ARRIVE guidelines for reporting animal research. PLoS Biol. 2010; 8:e1000412. 10.1371/journal.pbio.1000412. 20613859 PMC2893951

