# Correction: MYCN expression induces replication stress and sensitivity to PARP inhibition in neuroblastoma

**DOI:** 10.18632/oncotarget.28336

**Published:** 2023-01-12

**Authors:** David King, Xiao Dun Li, Gilberto S. Almeida, Colin Kwok, Polly Gravells, Daniel Harrison, Saoirse Burke, Albert Hallsworth, Yann Jamin, Sally George, Simon P. Robinson, Christopher J. Lord, Evon Poon, Daniel Yeomanson, Louis Chesler, Helen E. Bryant

**Affiliations:** ^1^Academic Unit of Molecular Oncology, Sheffield Institute for Nucleic Acids (SInFoNiA), Department of Oncology and Metabolism, University of Sheffield, Sheffield, UK; ^2^Divisions of Clinical Studies and Cancer Therapeutics, The Institute of Cancer Research, Sutton, UK; ^3^Divisions of Radiotherapy & Imaging, The Institute of Cancer Research, Sutton, UK; ^4^The Children and Young People’s Unit, The Royal Marsden NHS Trust, Sutton, UK; ^5^CRUK Gene Function Laboratory and Breast Cancer Now Research Centre, The Institute of Cancer Research, London, UK; ^6^Sheffield Children’s Hospital, Western Bank, Sheffield, UK; ^#^Present address: Medical Research Council Cancer Unit, Hutchison/Medical Research Council Research Centre, University of Cambridge, Cambridge, UK; ^*^These authors contributed equally to this work

**This article has been corrected:** In Supplementary Figure 2, under the IMR-32 column, the 3rd row, first panel image is an accidental duplicate of the 2nd row, 3rd panel image. The 6th row, 2nd panel image is also an accidental duplicate of the 7th row, 2nd panel image. In Supplementary Figure 11A, the 1st row, 2nd panel image was accidentally lifted from a student report as an example of IMR32 cells treated with DMSO. The corrected figures, obtained using the original data, are shown below. The authors declare that these corrections do not change the results or conclusions of this paper.

Original article: Oncotarget. 2020; 11:2141–2159. 2141-2159. https://doi.org/10.18632/oncotarget.27329

**Supplementary Figure 2:**
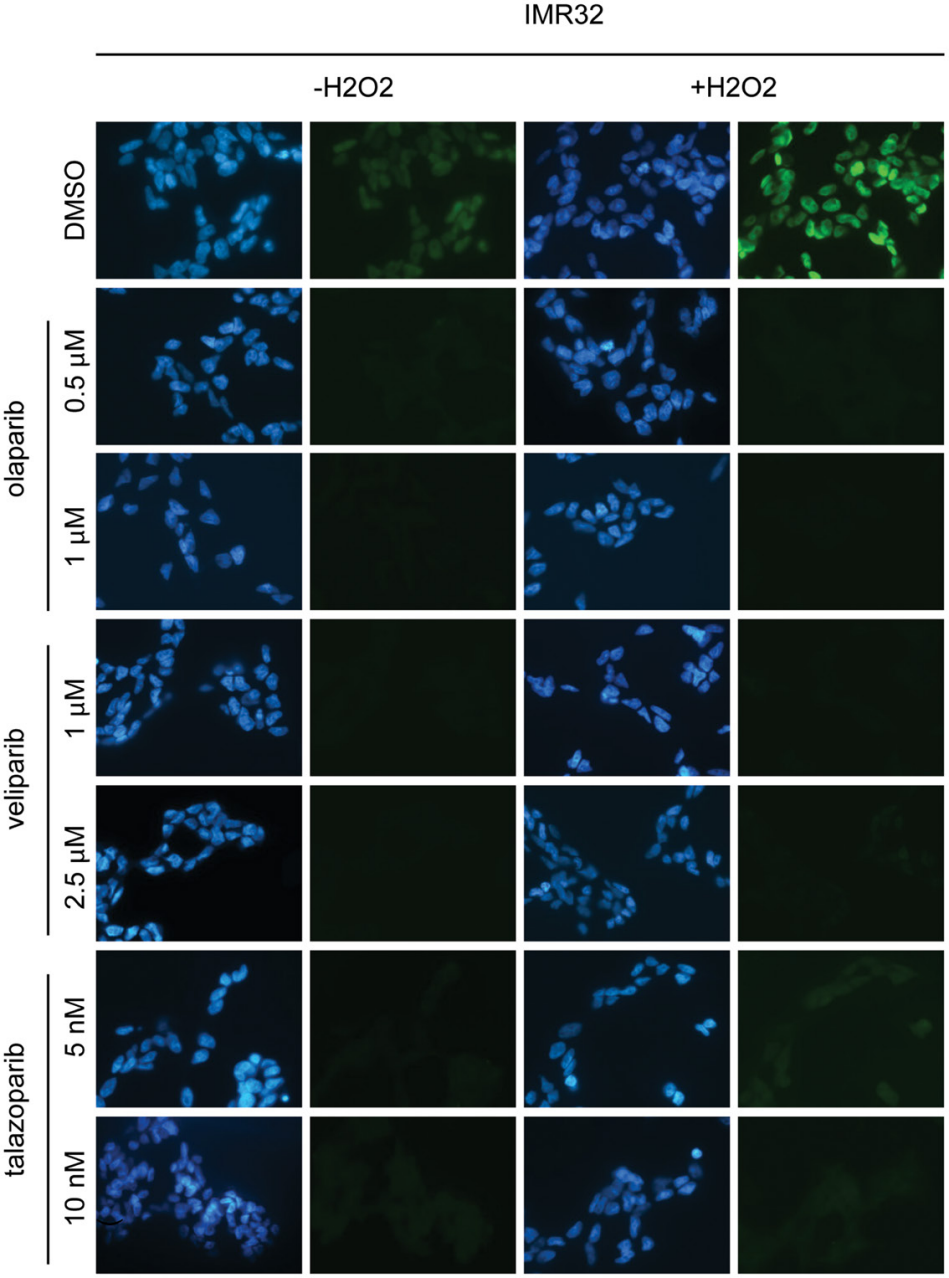
PARP inhibitors olaparib, veliparib and talazoparib inhibit PARP in NB cells. Detection of PAR by immunofluorescence in IMR-32 and Shep-1 NB cell lines. Cells were pre-treated for 16 hours at the concentrations of PARP inhibitors as indicated. Treating cells with 150 μM H_2_O_2_ resulted in much stronger PAR activity in vehicle-treated cells and allowed PARP inhibition to be more clearly demonstrated. PAR (green), DAPI (blue).

