# Supplementary material for: MYCN expression induces replication stress and sensitivity to PARP inhibition in neuroblastoma
Source: Oncotarget. 2020 Jun 9;11(23):2141–59. doi: 10.18632/oncotarget.27329 (PMC7289530; doi:10.18632/oncotarget.27329)
Supplement: Supplementary file 1 [file oncotarget-11-2141-s001.pdf]

MYCN expression induces replication stress and sensitivity to PARP inhibition in neuroblastoma

SUPPLEMENTARY MATERIALS FIGURES

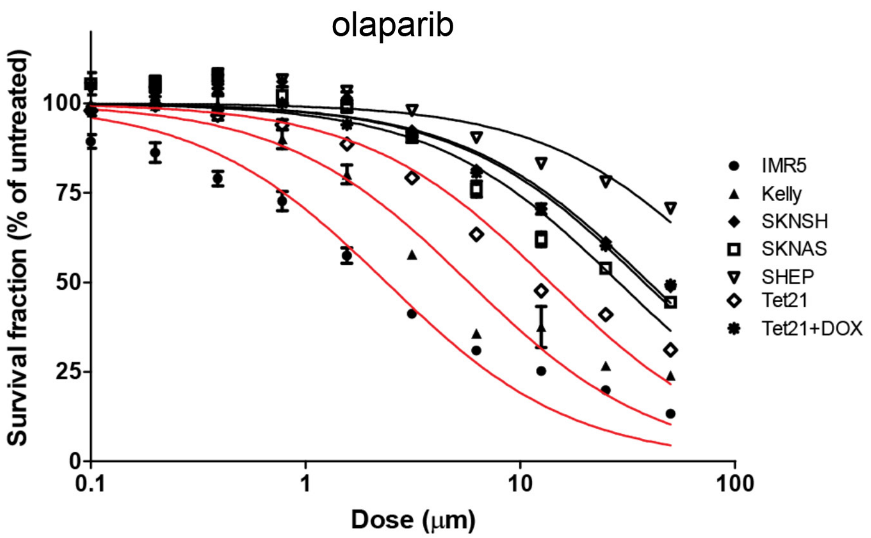

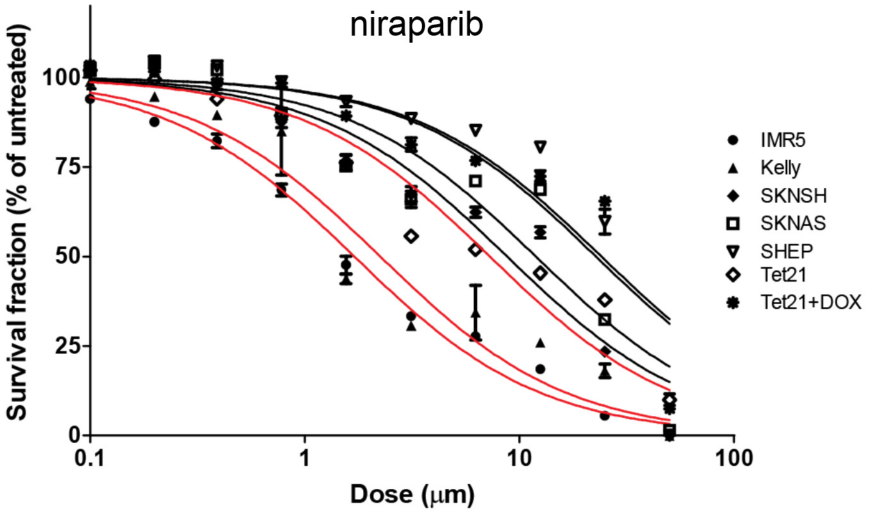

Supplementary Figure 1: PARP inhibitors olaparib and niraparib have lower GI_{50} values in *MYCN*-amplified and expressing NB cells. Relative cell survival was calculated using MTS assay after 72 h incubation in the PARP inhibitors olaparib and niraparib.

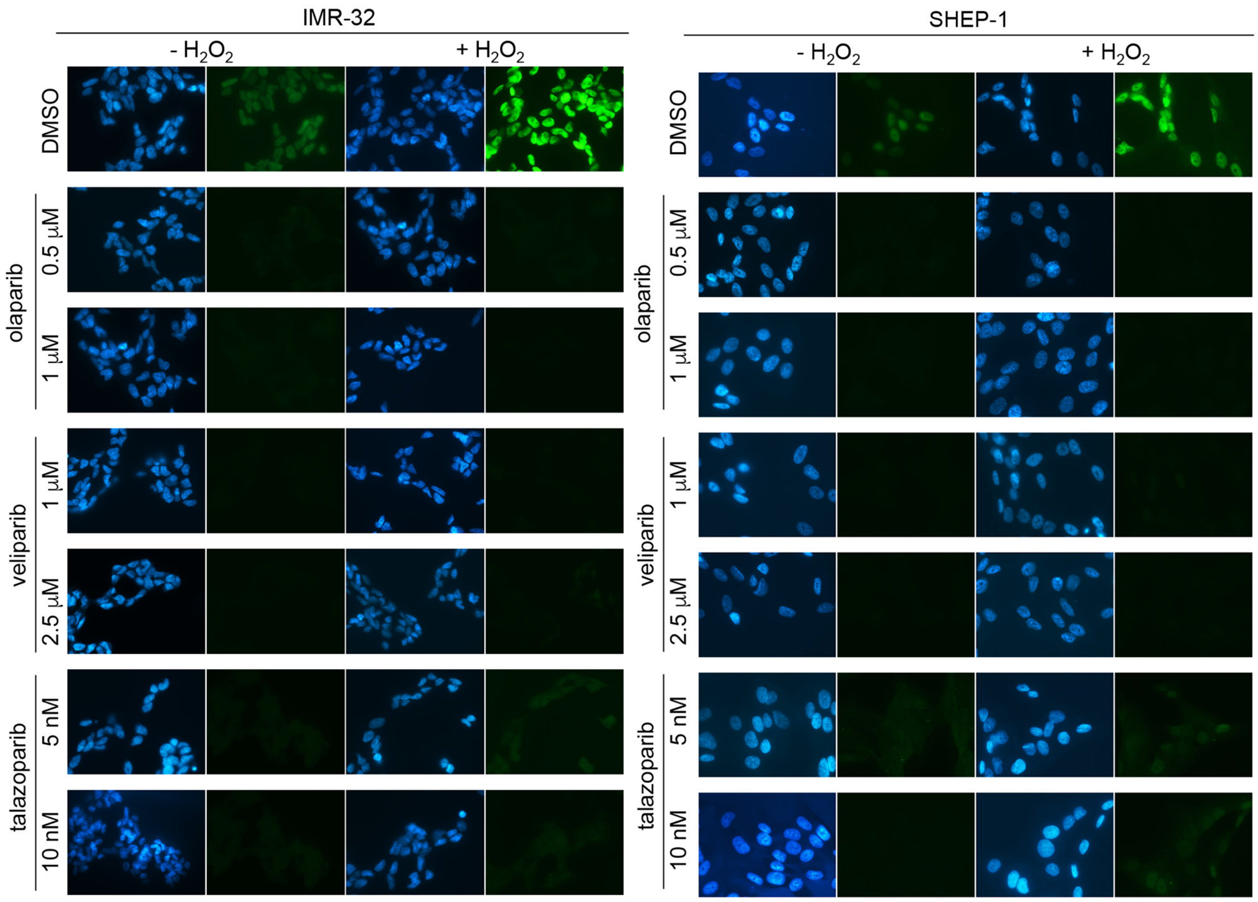

Supplementary Figure 2: PARP inhibitors olaparib, veliparib and talazoparib inhibit PARP in NB cells. Detection of PAR by immunofluorescence in IMR-32 and Shep-1 NB cell lines. Cells were pre-treated for 16 hours at the concentrations of PARP inhibitors as indicated. Treating cells with 150 µM H_2O_2 resulted in much stronger PAR activity in vehicle-treated cells and allowed PARP inhibition to be more clearly demonstrated. PAR (green), DAPI (blue).

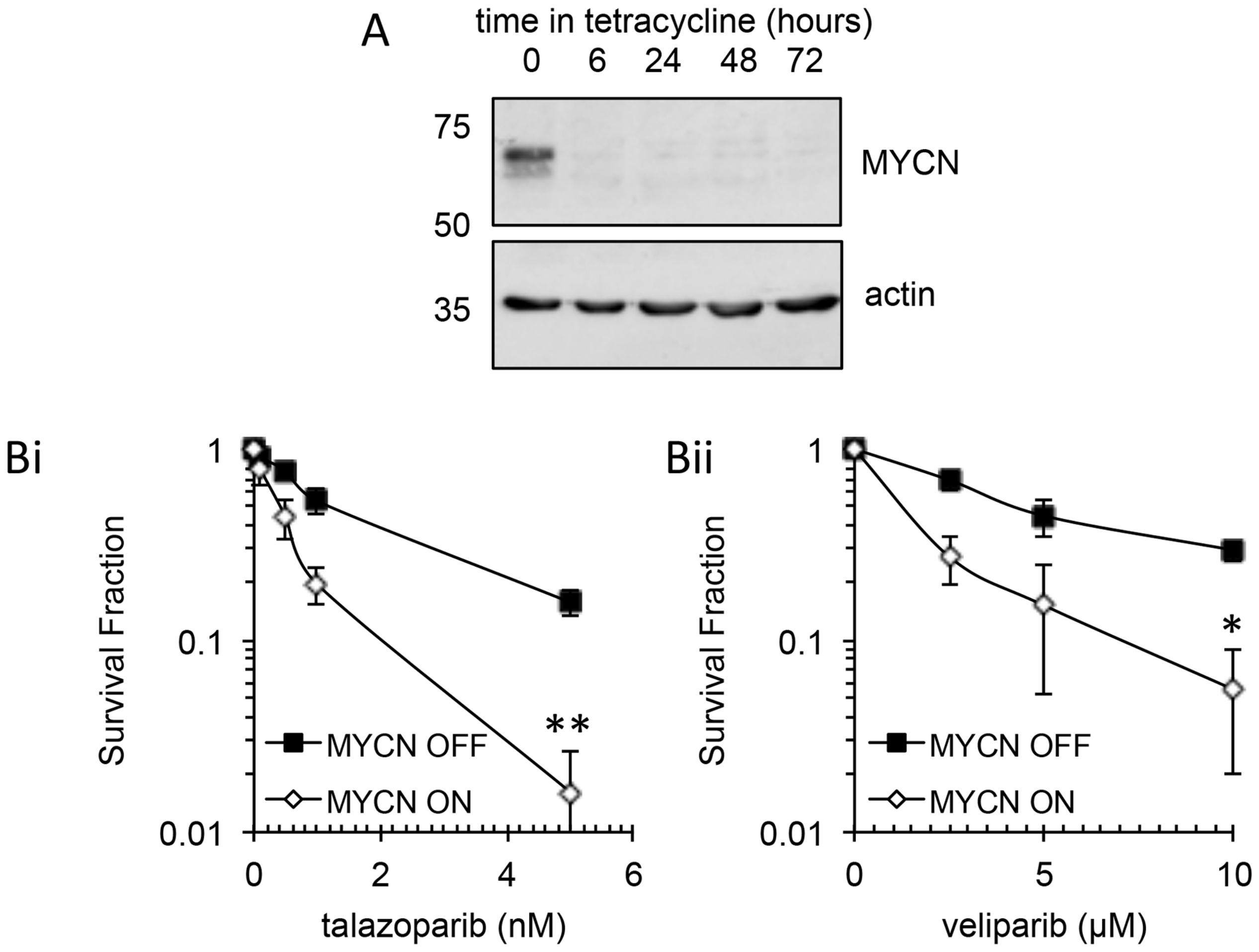

Supplementary Figure 3: (A) Validation of the SHEP-Tet21/N NB cell line with MYCN ON or OFF. Protein expression of MYCN after treatment of SHEP-Tet21/N cells with 1µg/ml tetracycline for the time indicated. **(B)** Survival fraction of SHEP-Tet21/N NB cell line with MYCN ON or OFF as measured by clonogenic survival assay 14 days post treatment with (i) talazoparib and (ii) veliparib. Statistical significance was calculated using the Student's t.test,. Mean and standard deviation of 3 independent repeats are shown. *= $p<0.05$ and **=$P<0.01$.

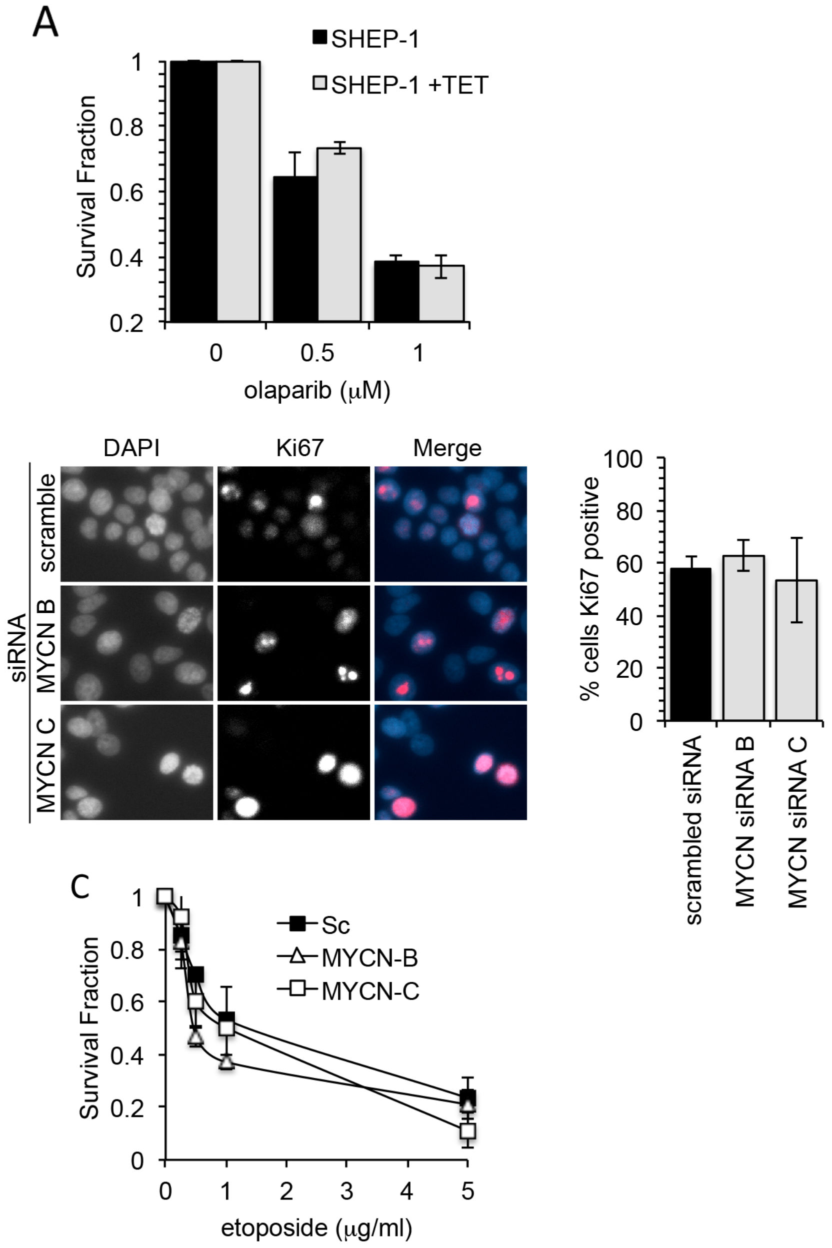

Supplementary Figure 4: **(A)** Tetracycline has no effect on sensitivity to olaparib in the parental SHEP-1 NB cell line. Cells were treated with olaparib at the doses indicated with and without 1μg/ml of tetracycline. Survival fraction was determined by clonogenic survival assay. Data = mean +/- SEM of 6 data sets over 3 independent repeats. **(B)** Proliferation and sensitivity to etoposide in IMR32 cells depleted of MYCN (i) Proliferation of IMR32 cells depleted of MYCN; live cells were Ki67 staining, (ii) cellular viability measured by MTT assay in the presence of increasing doses of etoposide. Data = mean +/-SD of 9 data sets over 3 independent repeats.

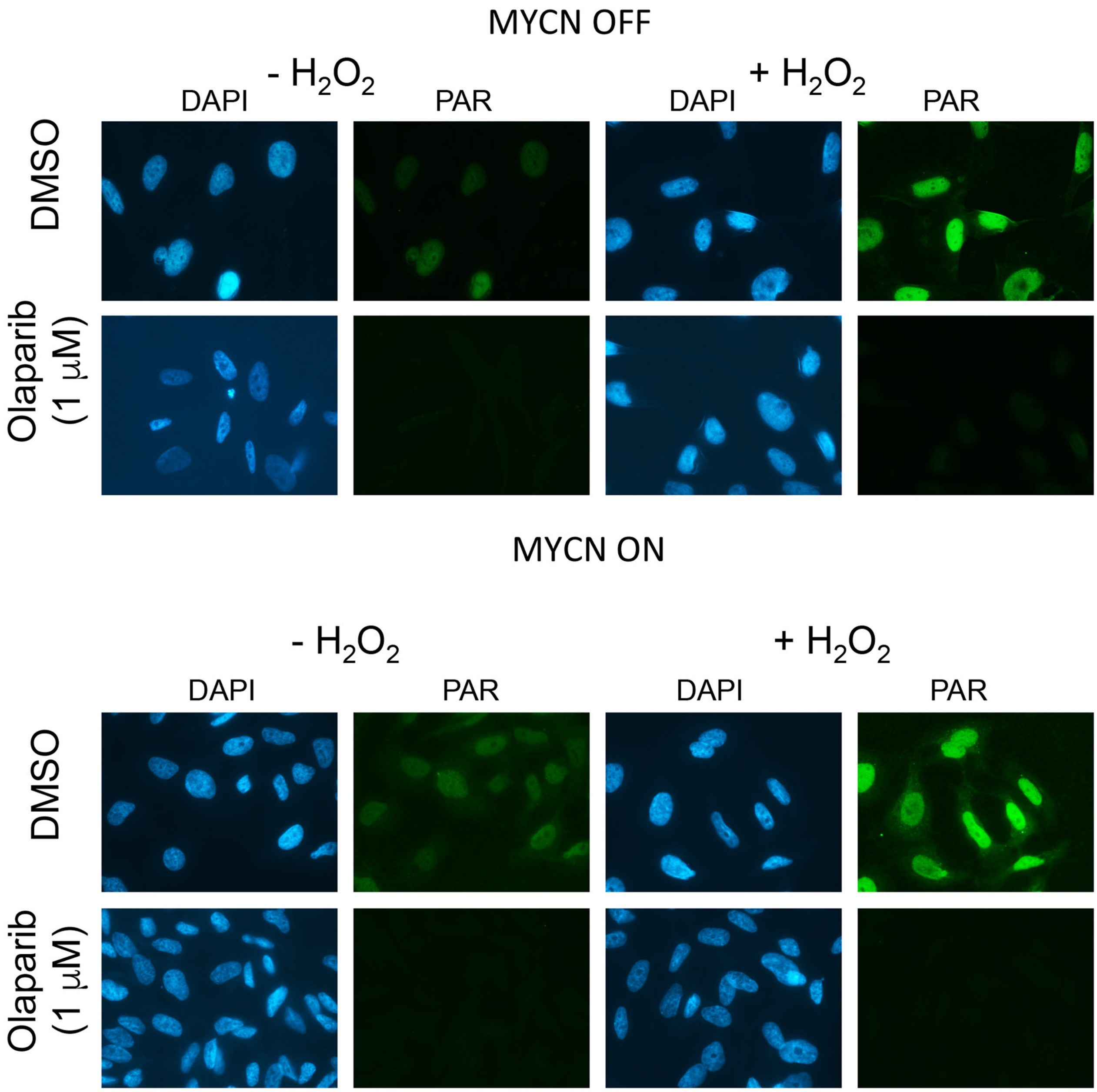

Supplementary Figure 5: Olaparib inhibits PARP in the SHEP-Tet21/N NB cell line with cell line regardless of MYCN expression. Olaparib inhibits detection of PAR by immunofluorescence in SHEP-Tet21/N NB cell line with MYCN ON or OFF. Cells were pre-treated for 16 hours with olaparib at the concentrations indicated. Treating cells with 150 μM H_2O_2 for 10 minutes on ice resulted in much stronger PAR activity in vehicle-treated cells and allowed PARP inhibition to be more clearly demonstrated. PAR (green), DAPI (blue).

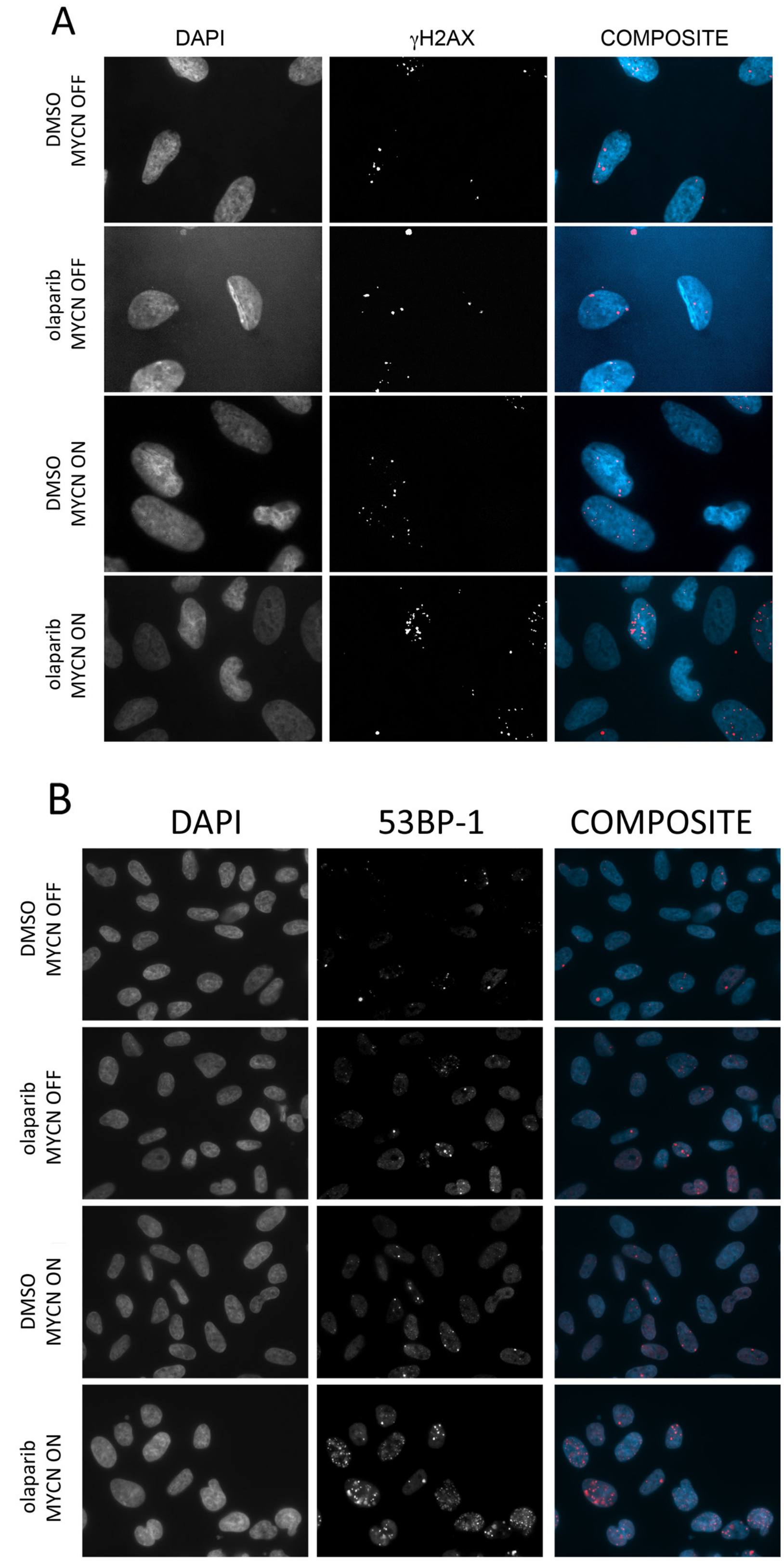

Supplementary Figure 6: Representative images of γH2AX and 53BP1 foci. **(A)** Representative images of γH2AX foci (red) co-stained with DAPI (blue) for the conditions indicated. **(B)** Representative images of 53BP-1 foci (red) co-stained with DAPI (blue) for the conditions indicated. In all cases cells were treated with 1μM olaparib for 16 hours.

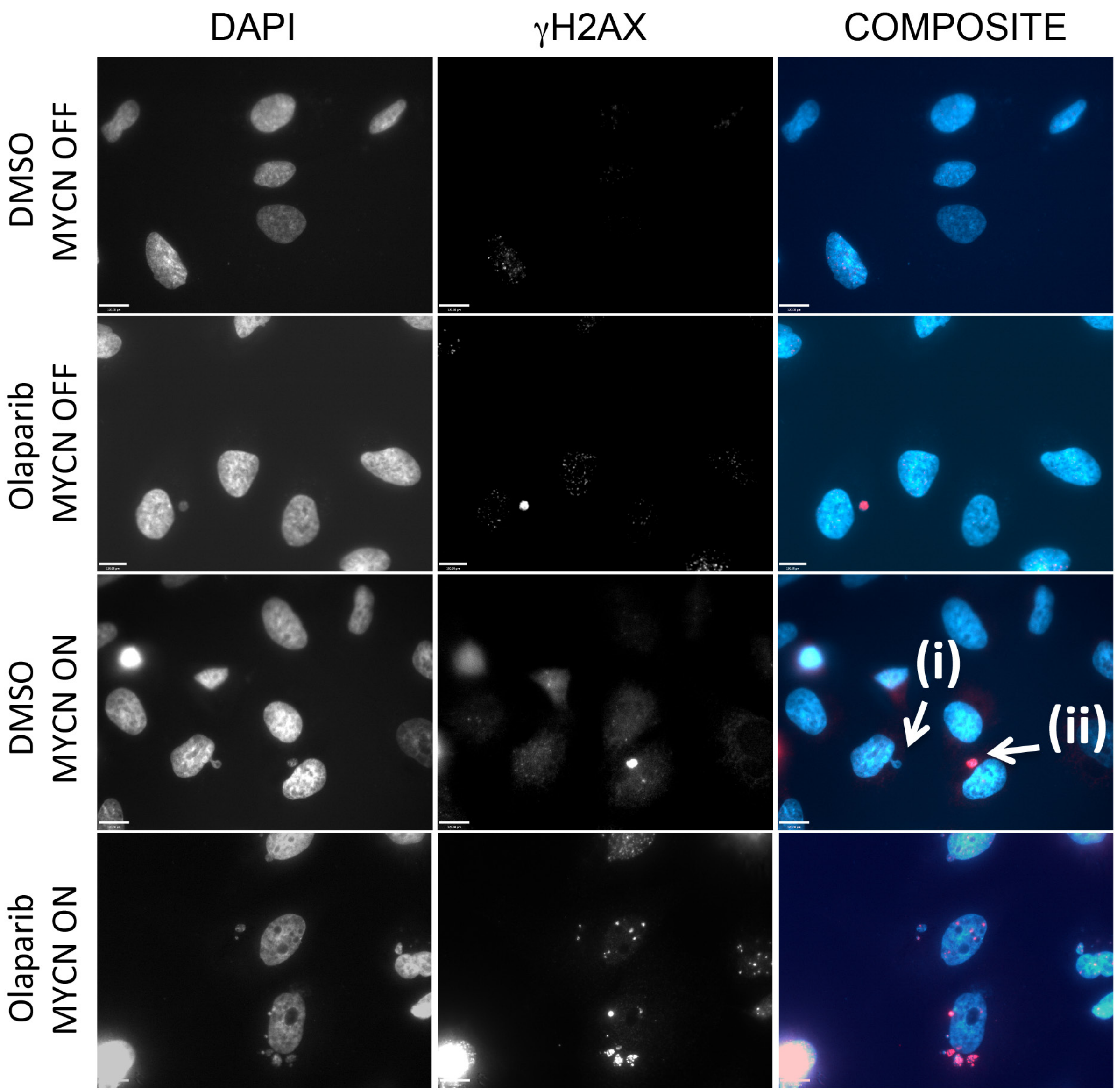

Supplementary Figure 7: Representative images of γH2AX positive MN. Representative images depicting (i) γH2AX negative and (ii) γH2AX positive MN for the conditions indicated. Cells were treated with 1μM olaparib for 16 hours.

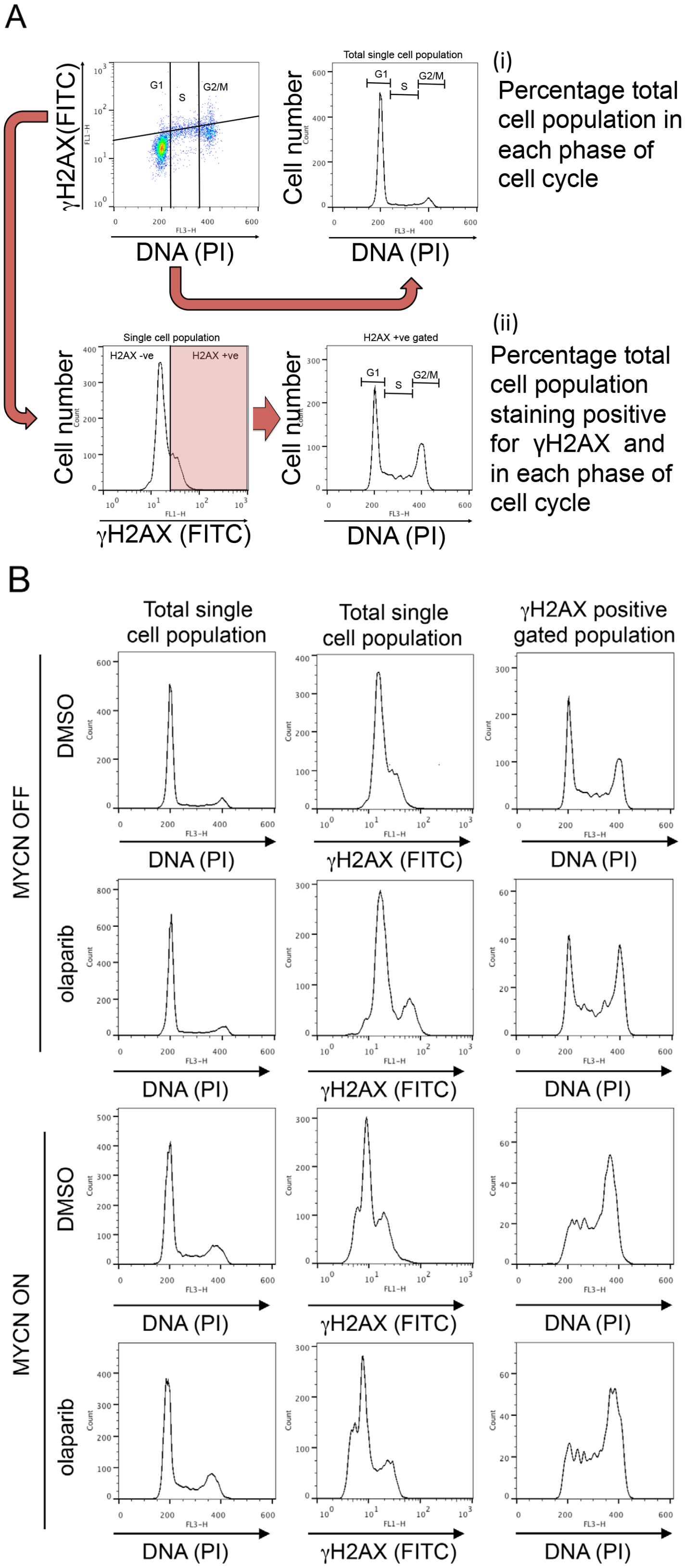

Supplementary Figure 8: Representative images for detection of γH2AX combined with differential staining of cellular DNA using flow cytometry. Cells were treated as indicated then fixed, γH2AX staining was performed using γH2AX antibody and cell cycle phase detected with propidium iodide. **(A)** The percentage of the total cell population in each phase of the cell cycle was calculated for (i) all cells and (ii) γH2AX positive cells. Example images are shown for one condition to illustrate how gating was performed. **(B)** SHEP-Tet21/N cells with MYCN ON or OFF were treated with 5 µM olaparib for 10 hours and compared with vehicle-treated controls. Representative images of the total cell cycle profile, γH2AX staining and the cell cycle profile in γH2AX positive cells are shown for each condition.

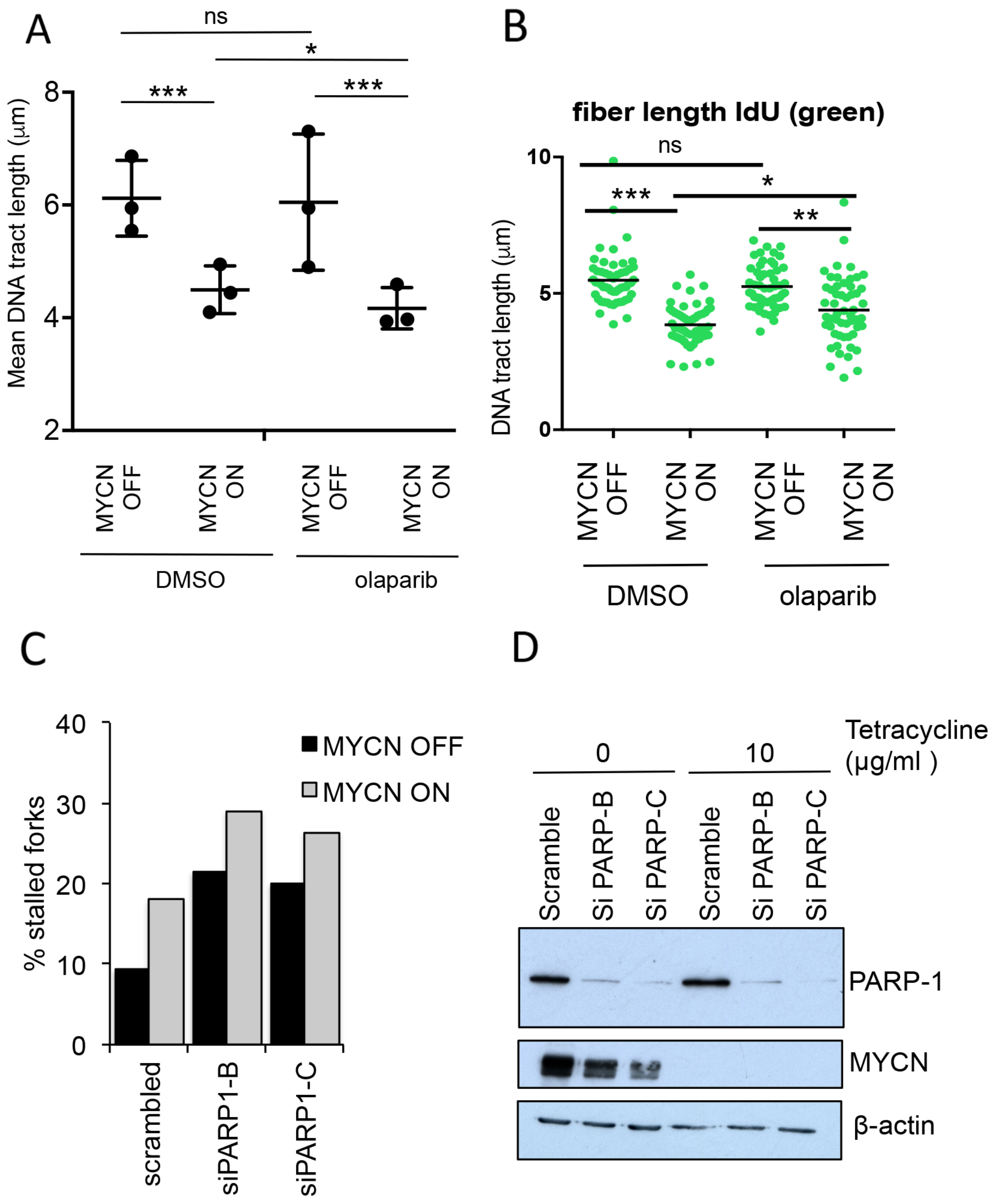

Supplementary Figure 9: DNA Fibre length in SHEP-Tet21/N cells with MYCN OFF or ON after olaparib treatment and in vehicle-treated controls. **(A)** Mean and SEM of 3 independent repeats of CidU (red) fibres, **(B)** Data are pooled from three independent repeats of IdU (green) fibres. Statistical significance calculated by Mann-Whitney U test between the conditions indicated where *, **, *** represent $p < 0.05$, 0.01, and <0.0001 respectively. **(C)** % stalled forks in SHEP-Tet21/N cells with MYCN OFF or ON after siRNA mediated depletion of PARP1 using 2 independent siRNA and in scrambled non-targeting control. Number of forks counted = 80-100 for each condition **(D)** Western blot confirming PARP-1 depletion and MYCN expression.

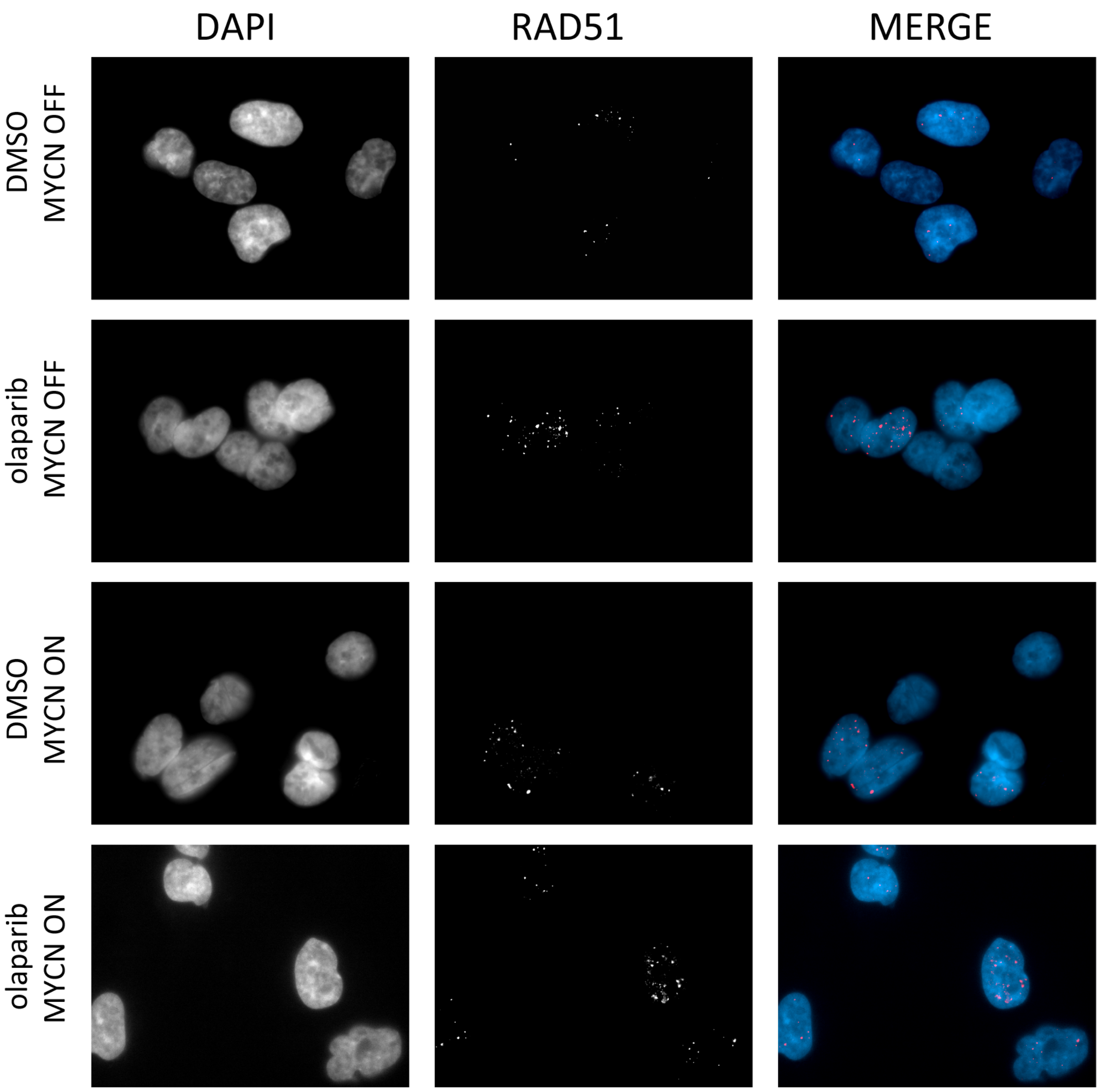

Supplementary Figure 10: Representative images of RAD51 foci. Representative images of RAD51 foci (red) co-stained with DAPI (blue) for the conditions indicated. In all cases cells were treated with 1µM olaparib for 16 hours.

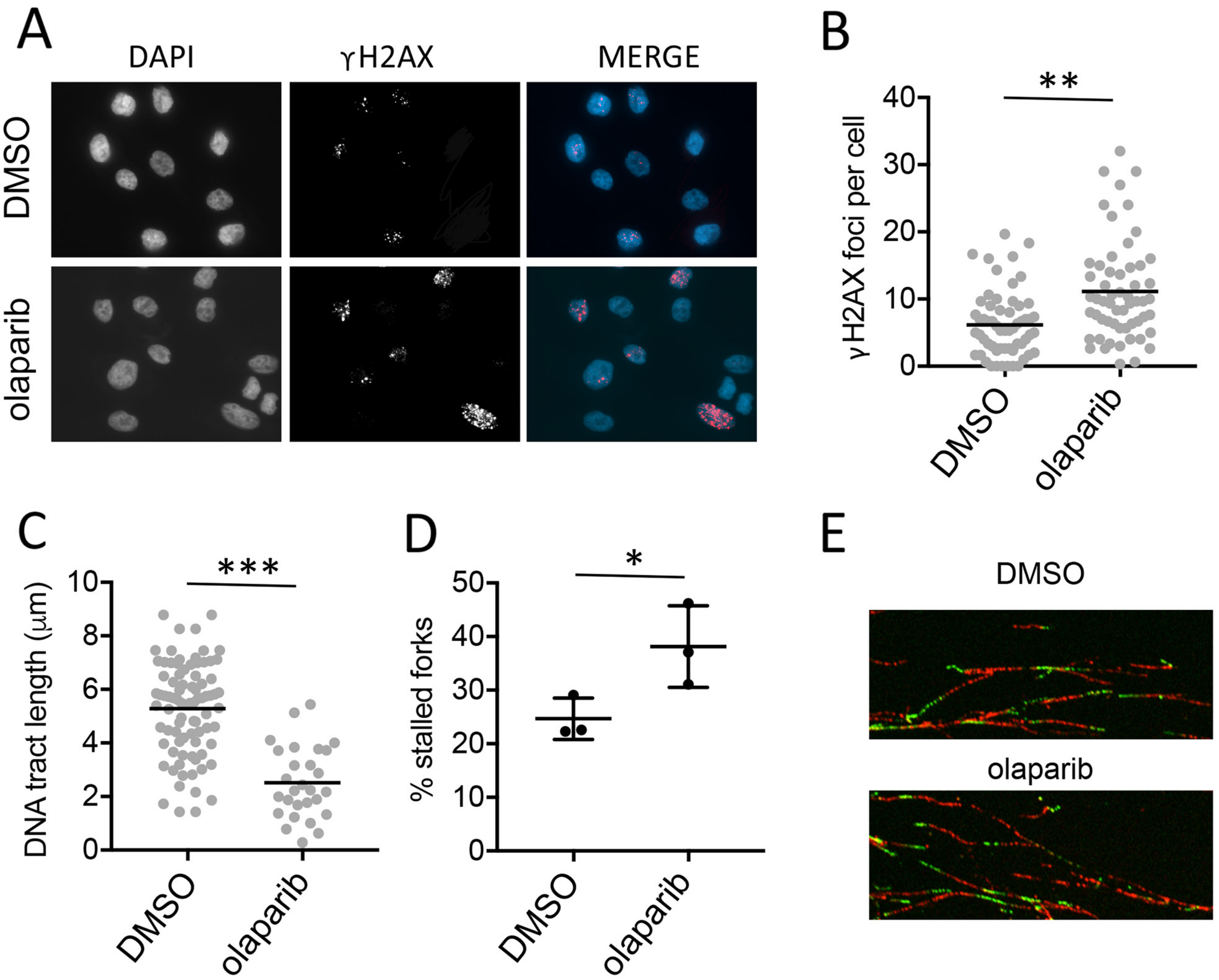

Supplementary Figure 11: DNA repair foci and Fibre assays in IMR32 cells. (A) Representative images depicting γH2AX for the conditions indicated. **(B)** Number of γH2AX foci/cell in SHEP-Tet21/N cells with MYCN ON and OFF 16 hours post treatment with 1 µM olaparib or DMSO control. Data shown are pooled from three independent repeats, for each repeat n>50 cells. Statistical significance was calculated using the Mann-Whitney U test. **(C)** DNA fibre length (µm) (CIdU), Statistical significance was calculated using the Mann-Whitney U test. **(D)** Percentage fork stalling, calculated as a % of CIdU only labelled tracts (red) from continuous forks (CIdU (red) and IdU (green) labelled tracts). Statistical significance was calculated using Student's T.test. **(E)** representative images.

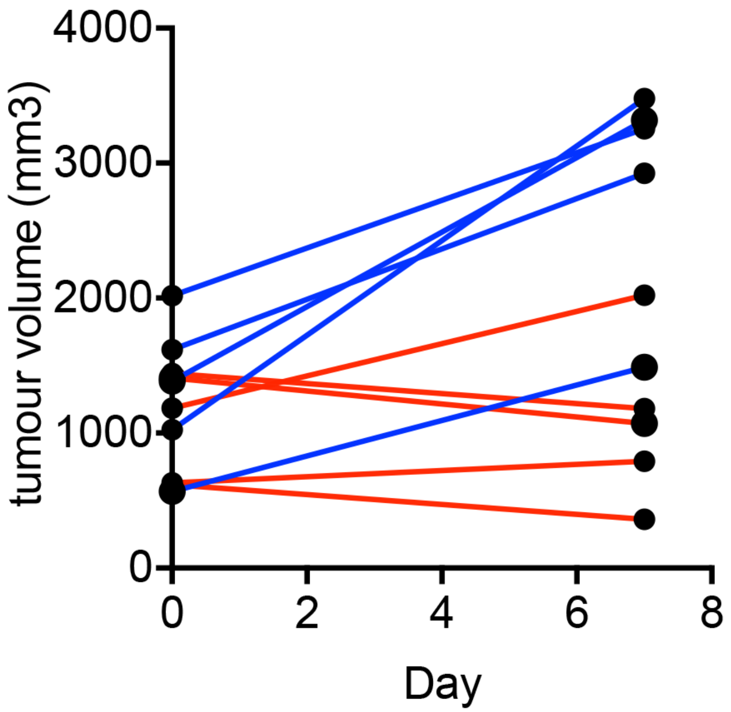

Supplementary Figure 12: Change in tumour volume following daily treatment of Th-MYCN mice with vehicle or 50mg/kg olaparib over 7days. Each mouse is represented with start weight and end weight plotted. Blue = control. Red = 50mg/kg olaparib.

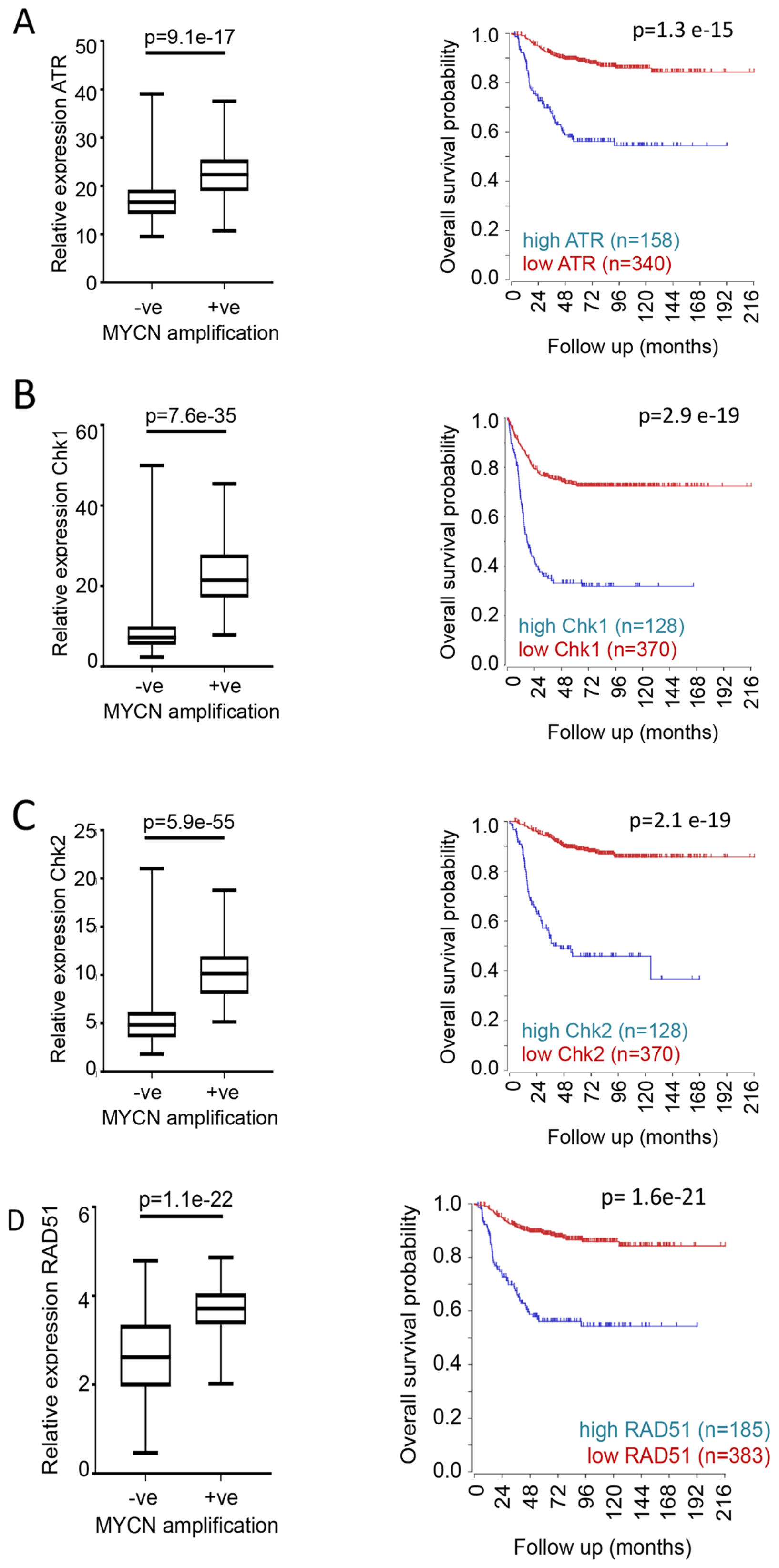

Supplementary Figure 13: High expression of key DDR genes correlates with a worse prognosis and *MYCN* status in patients with NB. **(A)** ATR **(B)** Chk1 **(C)** Chk2 **(D)** Rad51 expression all correlate with survival in NB and are higher in *MYCN*-amplified disease. For each gene, Kaplan-Meier curves demonstrating overall survival are shown for either high or low expression with p-values calculated using the log-rank test. Box-plots of gene expression are also shown with cases split according to *MYCN* status and p-values calculated using the one-way ANOVA. Analysis performed on cohort SEQC (n=493). Data obtained from the R2 platform (http://r2.amc.nl). ***(Continued)***

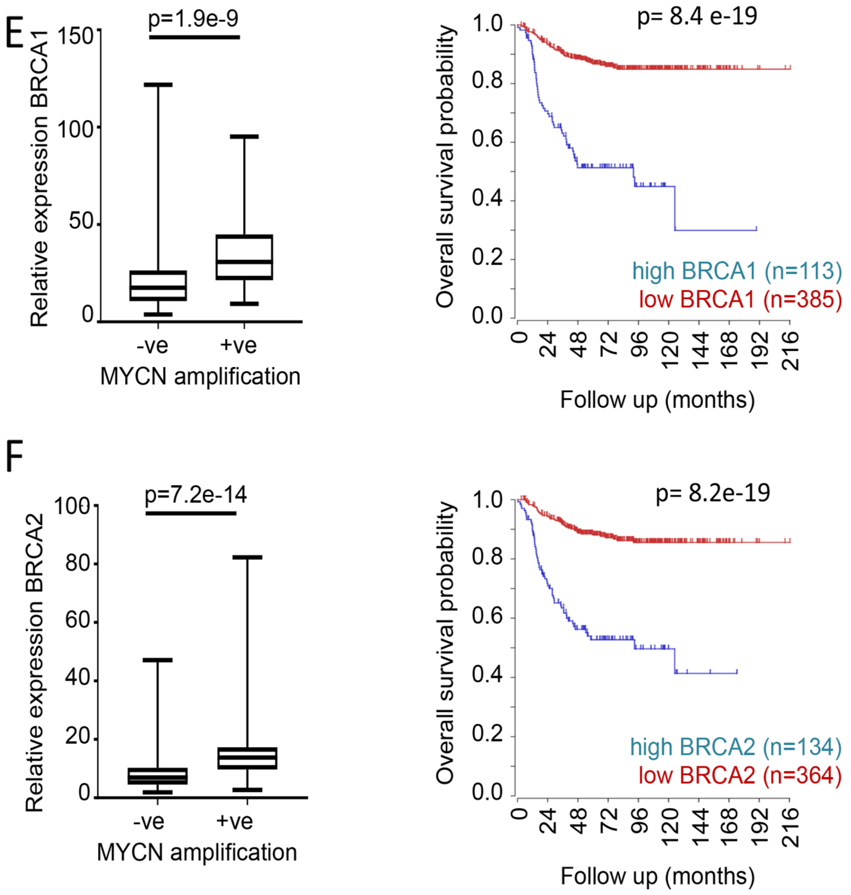

Supplementary Figure 13 (*Continued*): High expression of key DDR genes correlates with a worse prognosis and *MYCN* status in patients with NB. (E) BRCA1 and **(F)** BRCA2 expression all correlate with survival in NB and are higher in *MYCN*-amplified disease. For each gene, Kaplan-Meier curves demonstrating overall survival are shown for either high or low expression with p-values calculated using the log-rank test. Box-plots of gene expression are also shown with cases split according to *MYCN* status and p-values calculated using the one-way ANOVA. Analysis performed on cohort SEQC (n=493). Data obtained from the R2 platform (http://r2.amc.nl)
